# Ethanol infusion of the vein of marshall-based strategies for persistent atrial fibrillation: a systematic review and meta-analysis of randomized trials

**DOI:** 10.1093/europace/euag069

**Published:** 2026-04-02

**Authors:** Harroop Bola, Thalia Melamed, Mohamed Allaf, Joshua J Hon, Gayathri Giritharan, Amar Rai, Kiran Haresh Kumar Patel, Fu Siong Ng

**Affiliations:** Faculty of Medicine, Imperial College London, South Kensington Campus London, London SW7 2AZ, UK; London Northwest Healthcare NHS Trust, Department of Integrated Medicine, Northwick Park Hospital, London HA1 3UJ, UK; Faculty of Medicine, Imperial College London, South Kensington Campus London, London SW7 2AZ, UK; London Northwest Healthcare NHS Trust, Department of Integrated Medicine, Northwick Park Hospital, London HA1 3UJ, UK; Chelsea and Westminster Hospital NHS Foundation Trust, Department of Internal Medicine, London, UK; Faculty of Medicine, Imperial College London, South Kensington Campus London, London SW7 2AZ, UK; Faculty of Medicine, Imperial College London, South Kensington Campus London, London SW7 2AZ, UK; Faculty of Medicine, Imperial College London, South Kensington Campus London, London SW7 2AZ, UK; Department of Internal Medicine, University of Pennsylvania, Philadelphia, PA, USA; London Northwest Healthcare NHS Trust, Department of Integrated Medicine, Northwick Park Hospital, London HA1 3UJ, UK; National Heart and Lung Institute, Imperial College London, London, UK; Faculty of Medicine, Imperial College London, South Kensington Campus London, London SW7 2AZ, UK; Chelsea and Westminster Hospital NHS Foundation Trust, Department of Internal Medicine, London, UK; National Heart and Lung Institute, Imperial College London, London, UK; Department of Cardiology, Imperial College Healthcare NHS Trust, London, UK

**Keywords:** Atrial fibrillation, Catheter ablation, Persistent atrial fibrillation, Vein of Marshall, Ethanol infusion, Substrate modification

## Abstract

**Aims:**

Pulmonary vein isolation (PVI) alone achieves modest arrhythmia freedom in persistent atrial fibrillation (PeAF). Ethanol infusion of the vein of Marshall (EIVOM) overcomes heat-sink effects, facilitating mitral isthmus (MI) block, and may represent an effective adjunctive ablation strategy. We aimed to quantify the efficacy and safety of EIVOM through a meta-analysis of randomized controlled trials (RCTs).

**Methods and results:**

Systematic review of MEDLINE, Web of Science, and PubMed identified 5 RCTs enrolling 1179 patients (602 EIVOM, 577 control). The primary endpoint was 12-month freedom from any atrial arrhythmia. Random-effects models generated risk ratios (RRs) with 95% confidence intervals (CI). Time-to-event data were pooled using a generic inverse-variance approach to derive hazard ratios (HR). EIVOM-based strategies improved freedom from any arrhythmia (RR 1.16, 95% CI 1.04–1.29; *P* < 0.001; number needed to treat (NNT) = 10) and from atrial fibrillation (RR 1.11, 95% CI 1.05–1.18; *P* < 0.001; NNT = 13). Time-to-event analysis demonstrated a sustained reduction in recurrence hazard (HR 0.72, 95% CI 0.64–0.81; *P* = 0.003; *I*^2^ = 0%). Repeat ablation was reduced (RR 0.61; *P* = 0.009). Fluoroscopy time increased (+9.08 min; *P* = 0.007), while major complications were comparable (2.5% vs. 2.8%; *P* = 0.47). Trial sequential analysis confirmed that the cumulative Z-curve crossed the monitoring boundary for benefit for the primary endpoint, indicating that the available evidence is sufficient.

**Conclusion:**

In PeAF, EIVOM-based ablation strategies significantly improve 12-month arrhythmia-free survival and reduce repeat procedures without increasing major adverse events. However, the observed benefit reflects composite ablation sets rather than ethanol infusion in isolation, and the predominance of high-volume expert centres may limit generalizability.

## Graphical Abstract

**Graphical Abstract:**
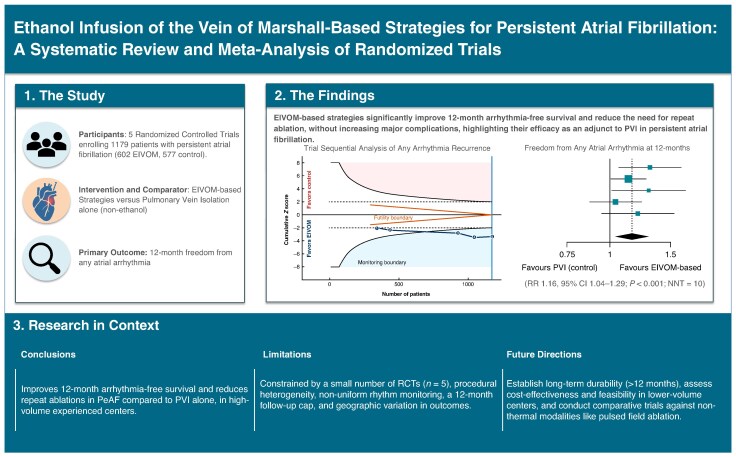

## Introduction

While pulmonary vein isolation (PVI) serves as the cornerstone of catheter-based ablative management,^1^ its efficacy as a standalone strategy in persistent atrial fibrillation (PeAF) populations is modest, with single-procedure arrhythmia-free 12-month survival rates plateauing at 50–70%.^2,3^ This attrition is frequently attributed to the presence of extra-pulmonary substrates that sustain the arrhythmia, specifically non-PV triggers and anatomical re-entrant circuits.^4–6^ Among these, the mitral isthmus (MI) is an important yet challenging ablation target.^7^ Achieving durable bidirectional block across this region is often impeded by the heat-sink effect of blood flow within the coronary sinus (CS) and the presence of the epicardial Vein of Marshall (VoM), which can bridge the ablation line and facilitate residual conduction.^8–10^

The VoM, an oblique vein and remnant of the left superior vena cava, serves as a unique arrhythmogenic structure. It not only provides an epicardial bypass for MI conduction but is also richly innervated by parasympathetic ganglia and sympathetic fibres that contribute to autonomic dysregulation. Conventional endocardial radiofrequency (RF) ablation frequently fails to transmurally affect these epicardial structures due to the insulating properties of epicardial fat.^11–14^ Consequently, the Marshall bundle remains a common source of reconnection and perimitral flutter recurrence following standard ablation sets.^15^ Ethanol infusion into the Vein of Marshall (EIVOM) offers a mechanism-based adjunct to standard PVI. By creating a localized chemical ablation, EIVOM eliminates Marshall bundle potentials, denervates the surrounding autonomic ganglia, and facilitates the creation of a transmural lesion across the MI.^16,17^

Recent randomized controlled trials (RCTs), including VENUS and Marshall-Plan, have suggested that incorporating EIVOM into PeAF ablation strategies significantly reduces arrhythmia recurrence compared with PVI alone.^18–22^ While these trials have collectively signaled a therapeutic advantage over isolated PVI, variations in study design, adjunctive lesion sets, and safety reporting have precluded a definitive consensus regarding the procedure’s broad implementation. Furthermore, the interpretation of the aggregate evidence has been hindered by the limitations of prior meta-analytic syntheses. Previous reviews have frequently included phenotypically heterogeneous populations, diluting the analysis with paroxysmal atrial fibrillation (AF) cohorts, and have relied heavily on observational and retrospective datasets susceptible to selection bias.^23,24^ Furthermore, these studies have often lacked a granular examination of procedural efficacy (e.g. acute block success) and specific safety profiles.^25^ To resolve ongoing clinical equipoise and provide granular data for procedural planning, we conducted a systematic review and meta-analysis restricted to RCTs. This study aims to quantify the incremental benefit of EIVOM-based strategies on long-term sinus rhythm maintenance and to rigorously assess the procedural efficacy and safety profile in the specific context of PeAF.

## Methods

### Study design and protocol registration

This systematic review and meta-analysis were conducted in strict accordance with the Preferred Reporting Items for Systematic Reviews and Meta-Analyses (PRISMA) 2020 guidelines and the Cochrane Handbook for Systematic Reviews of Interventions.^26,27^ The study protocol was prospectively registered in the International Prospective Register of Systematic Reviews (PROSPERO registration: CRD420251146539) before initiation of the literature search and data extraction.

### Literature search strategy

A comprehensive, systematic literature search was conducted across three electronic databases—MEDLINE (via Ovid), Web of Science Core Collection, and PubMed, from database inception through to 1st October 2025. The full search strategy is given in Supplementary material online, *Table S1*. No language, publication date, or geographical restrictions were imposed.

### Eligibility criteria

Study eligibility was determined using a structured Population, Intervention, Comparator, Outcome, and Study design framework.

#### Population

Studies were required to enroll adult patients (age ≥18 years) with symptomatic PeAF, defined according to established international consensus criteria (continuous AF >7 days’ duration or requiring cardioversion after ≥48 h). Exclusion criteria comprised paediatric populations, patients with paroxysmal AF exclusively, preclinical or animal investigations, and studies enrolling patients undergoing surgical or hybrid thoracoscopic ablation procedures.

#### Intervention

The intervention arm required catheter-based PVI with adjunctive EIVOM performed during the index ablation procedure. PVI could be achieved using RF or cryothermal energy modalities. Additional linear ablation lesion sets (MI, left atrial (LA) roof, cavotricuspid isthmus) were permissible. Studies employing staged ethanol infusion procedures, surgical VoM ligation, or alternative investigational substrate modification techniques without ethanol infusion were excluded.

#### Comparator

The control arm required PVI alone delivered via RF or cryothermal energy. Empirical linear lesion sets were permissible provided EIVOM was not performed. Comparators involving surgical ablation, hybrid procedures, or investigational VoM interventions were excluded.

#### Outcomes

Studies were required to report at least one prespecified primary efficacy endpoint procedural endpoint, or safety endpoint, with quantitative data presented separately for intervention and control arms. Minimum follow-up duration was 12 months for efficacy endpoints.

#### Study design

Inclusion was restricted to parallel-group RCTs published in peer-reviewed journals or presented as conference abstracts with sufficient extractable data. Observational studies, case series, editorials, narrative reviews, and animal or *in vitro* investigations were excluded.

### Outcome measures

#### Primary efficacy endpoints

Three co-primary efficacy endpoints were prespecified: (1) freedom from any atrial arrhythmia recurrence at 12 months (composite endpoint encompassing AF, atrial flutter recurrence (AFL), and atrial tachycardia (AT) >30 s duration following the blanking period); (2) freedom from AF recurrence at 12 months (defined as absence of AF episodes >30 s duration following a 90-day blanking period); and (3) freedom from AT or AFL at 12 months (defined as absence of organized atrial tachyarrhythmias >30 s duration following the blanking period).

#### Secondary procedural endpoints

Prespecified procedural endpoints included: (1) total procedure time from venous access to catheter removal; (2) total fluoroscopy duration; (3) total RF ablation time; (4) acute achievement of bidirectional MI conduction block; and (5) requirement for adjunctive or repeat ablation procedures.

#### Safety endpoints

The primary safety endpoint was the composite incidence of any procedure-related complications or adverse events occurring within 30 days of the index procedure or during the follow-up period. Secondary safety endpoints included: (1) incidence of major complications, defined according to the classification system reported by Derval et al^18^; (2) incidence of pericardial effusion or pericarditis; and (3) incidence of cardiac tamponade.

### Study selection and data extraction

All retrieved records were imported into systematic review management software (Covidence, Veritas Health Innovation, Melbourne, Australia). Following automated removal of duplicates (*n* = 239) and records triaged as ineligible (*n* = 1189), at least two independent reviewers (M.A., T.M., J.H., G.G.) screened titles and abstracts of the remaining 797 records for potential eligibility. Full-text review was conducted for 14 studies, of which five RCTs met all eligibility criteria and were included in the quantitative synthesis. Discordant eligibility determinations were adjudicated by a third independent reviewer (H.B.) through consensus discussion.

Data extraction was performed independently and in duplicate using a standardized, piloted electronic data extraction form. Extracted variables included study identifiers, methodological characteristics, population demographics, procedural details, and outcome data. For the Zhu et al. study,^21^ event data corresponding to survival outcomes were not explicitly tabulated; these data points were accurately derived from published Kaplan–Meier curves using the digitalizing software, WebPlotDigitizer (version 4.6).

### Quality assessment and risk of bias

Methodological quality and risk of bias of included trials were independently assessed by at least two reviewers (M.A., T.M., J.H., G.G.) using the revised Cochrane Risk of Bias tool for randomized trials (RoB-2).^28^ Discrepancies were resolved through consensus discussion with adjudication by a third reviewer (H.B., A.R.) when necessary.

### Statistical analysis

All statistical analyses were performed using R statistical software version 4.5.2 (R Foundation for Statistical Computing, Vienna, Austria) with the meta package version 7.0–0. For dichotomous outcomes, the relative risk (RR) and corresponding 95% confidence interval (CI) were calculated using the Mantel–Haenszel method. For continuous outcomes, the mean difference (MD) and 95% CI were estimated using the inverse variance method. Statistical significance was defined as *P* ≤ 0.05 for all analyses.

### Time-to-event analysis

To account for the temporal distribution of arrhythmia recurrence and evaluate treatment durability, a secondary time-to-event meta-analysis was performed. For trials not reporting hazard ratios (HRs) with corresponding 95% CIs, individual patient data were reconstructed from published Kaplan–Meier curves using the method described by Guyot et al.^29^ Study-specific HRs and standard errors were subsequently derived using Cox proportional hazards regression applied to the reconstructed datasets. Log-transformed HRs were pooled using a random-effects generic inverse-variance model. Between-study variance was estimated using restricted maximum likelihood, and the Hartung–Knapp–Sidik–Jonkman adjustment was applied to provide conservative inference given the limited number of studies. Statistical significance was defined as a two-sided *P* value ≤0.05.

### Heterogeneity assessment and model selection

Between-study heterogeneity was assessed using the Cochran *Q* statistic (significance threshold *P* < 0.10) and the *I*^2^ statistic. *I*^2^ values were interpreted according to Cochrane Handbook thresholds: 0–40% indicating minimal heterogeneity, 30–60% moderate, 50–90% substantial, and 75–100% considerable heterogeneity.^27^

A random-effects model was prespecified for all primary analyses to account for both within-study and between-study variability. This conservative approach was selected *a priori*, given anticipated clinical heterogeneity in patient populations, adjunctive ablation strategies, and follow-up protocols across included studies.

For meta-regression, each eligible predictor was assessed individually through simple meta-regression, given the limited number of studies (*n* = 5). Predictor variables included baseline patient characteristics, procedural parameters, and study-level metrics. Random-effects meta-regression was performed using restricted maximum likelihood estimation. Multivariate meta-regression was not performed due to the limited number of studies.

For clinically significant dichotomous efficacy outcomes, the number needed to treat (NNT) was calculated to provide absolute risk measures that complement RR estimates.^30^ NNT represents the number of patients who would need to receive the EIVOM strategy rather than PVI alone to achieve one additional beneficial outcome event. The full calculation methodology is provided in the Supplementary Appendix. Trial sequential analysis (TSA) was performed to assess conclusiveness and risk of random error, calculating diversity-adjusted required information sizes (RIS) based on pooled control event rates and a conservative 20% RR reduction.^31^

### Publication bias and sensitivity analyses

Publication bias was assessed using funnel plot visualization. Given the limited number of included studies (*n* = 5), formal statistical tests for publication bias (e.g. Egger's regression) were not performed due to insufficient power. Robustness of pooled estimates was evaluated using leave-one-out sensitivity analysis.

Results were displayed graphically using forest plots depicting study-level and pooled effect sizes with corresponding 95% CIs.

## Results

**Figure 1:**
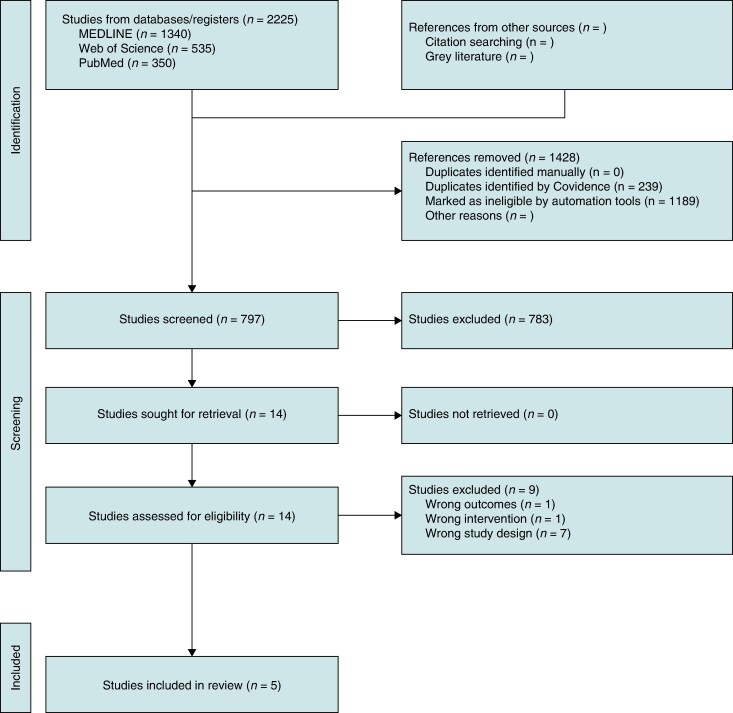
PRISMA flowchart.

The initial systematic search yielded 2225 records. Following the removal of duplicates and preliminary screening of titles and abstracts, 14 full-text articles were assessed for eligibility. Ultimately, 5 RCTs enrolling a total of 1179 patients were identified for inclusion in the quantitative synthesis.^18–22^ The study selection process is detailed in the PRISMA flow diagram (*Figure 1*). These trials consisted of two single-centre and three multicentre investigations, with all studies comparing EIVOM-based strategies against PVI alone (or PVI plus standard linear ablation) in patients with PeAF.

### Study characteristics

The population for this meta-analysis comprised a total of 1179 patients: 602 receiving EIVOM-based ablation and 577 treated with PVI without ethanol infusion. Out of the 1179 patients, 874 were male (74.1%), with an overall mean age of 63.2 years. The mean LA diameter was 44.2 mm, and the average duration of known AF pre-procedure was 16.4 months. Baseline data in the population and the design of the selected trials are summarized in Supplementary material online, *Table S2* and *Table 1*.

**Table 1:** Characteristics of included randomized controlled trials

First author, year (study acronym or registry)	Country	No. Patients	Ablation procedure	PVI group ablation technique	Marshall plan strategy	Follow-up, months	Rhythm Monitoring Protocol
**Derval, 2025, Single-centre Marshall-Plan** ^18^	France	118	Radiofrequency	CPVI	EIVOM + PVI + empirical linear ablation [anatomic isthmus blocks, (mitral, dome and CT Isthmuses)]	12	**Scheduled:** Weekly transtelephonic electrocardiogram monitors and Holter monitoring at 3−, 6−, 9−, and 12-month clinic visits.
**Sang, 2025,** **Multicentre** **PROMPT-AF** ^19^	China	495	Radiofrequency	CPVI	EIVOM + PVI + linear ablation	12	**Scheduled:** Wearable single-lead ECG patches worn for 24 h every week for the entire 12-month follow-up period. **Adjunctive:** Standard Holter monitoring and symptom-triggered 12-lead ECGs performed as needed.
**Valderrábano**,**2020,** **Multicentre** **VENUS**^20^	USA	343	Radiofrequency	CPVI	EIVOM + PVI + MI ± Carina/posterior wall/complex potentials	12	**Scheduled:** 30-day continuous external monitors (MediLynx) at months 6 and 12; 12-lead ECGs at 1−, 3−, 6−, 9−, and 12-month clinic visits. **Adjunctive:** Interrogation of implanted devices (pacemakers/ICDs) continuously monitored and utilized if already present.
**Zhu,2025** **Single centre** ^21^	China	134	Radiofrequency	CPVI	EIVOM + CPVI + modified linear ablation (left atrial PWI, linear ablation of MI, LAI-CS AND SVCI	14	**Scheduled:** 24-hour Holter monitoring at 1, 3, and 6 months; prolonged 7-day Holter monitoring at 12 months. **Adjunctive:** 12-lead ECGs performed at all standard outpatient clinic visits.
**Zuo, 2024** **Single centre** ^22^	China	89	Radiofrequency	RFCA (CPVI, MI line, intra CS and CTI + mapping guided ablation	CPVI + EIVOM + linear ablation (MI, CS, CTI, LA roofline)	12	**Scheduled:** 12-lead ECGs and 24-h Holter monitoring performed at 3−, 6−, and 12-month follow-up visits. **Adjunctive:** Symptom-driven assessment for documented arrhythmias (triggering repeat procedures if symptomatic/documented).

**Abbreviations:** AF, atrial fibrillation; CPVI, circumferential pulmonary vein isolation; CS, coronary sinus; CTI, cavotricuspid isthmus; EIVOM, ethanol infusion of the vein of Marshall; LA, left atrial; LAI-CS, left atrial intima adjoining coronary sinus; MI, mitral isthmus; NR, not reported; PVI, pulmonary vein isolation; PWI, posterior wall isolation; RFCA, radiofrequency catheter ablation; SVCI, superior vena cava isolation.

### Quality assessment

**Figure 2:**
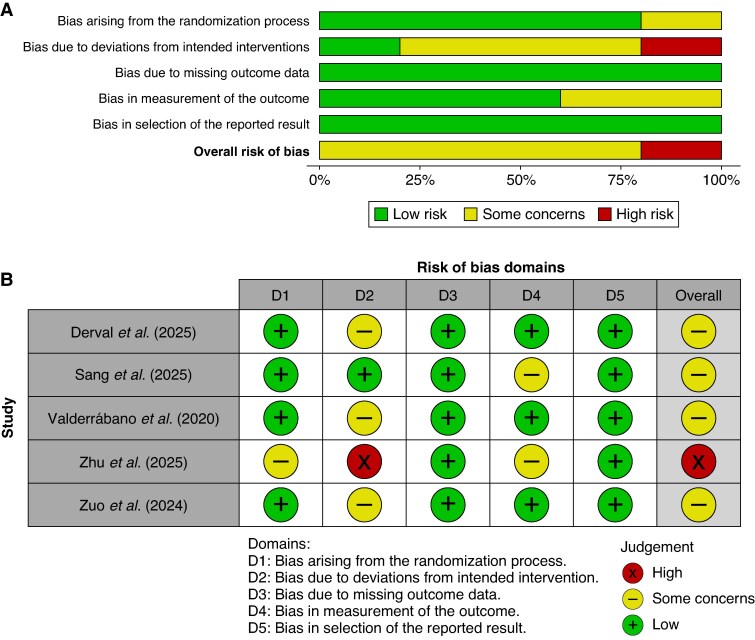
Risk of bias assessment of included randomized clinical trials. Assessment was performed using the Cochrane Risk of Bias 2 (RoB-2) tool. (*A*) Weighted summary plot illustrating the proportion of studies with low risk, some concerns, or high risk of bias for each domain. (*B*) Study-level ‘traffic light’ plot detailing judgments for each domain across the 5 included trials. Abbreviations: D1 = Randomization process; D2 = Deviations from intended interventions; D3 = Missing outcome data; D4 = Measurement of the outcome; D5 = Selection of the reported result.

Risk of bias assessment using the RoB-2 tool revealed acceptable methodological quality overall (*Figure 2)*. Four trials demonstrated some concerns, primarily attributable to the inherent open-label design of procedural interventions and operator-dependent procedural execution. One trial (Zhu et al. 2025) was rated as high risk of bias in the deviations from intended interventions domain due to protocol deviations from failed EIVOM in a subset of patients.^21^ However, all trials employed appropriate randomization procedures, maintained complete follow-up, and utilized objective electrophysiological endpoints, including systematic electrocardiographic and Holter monitoring. These methodological strengths mitigate concerns regarding detection bias and support the validity of pooled estimates.

### Primary efficacy outcomes

#### Freedom from any arrhythmia

**Figure 3:**
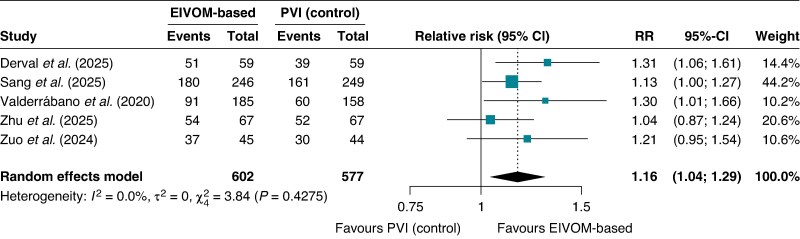
Forest plot of freedom from any atrial arrhythmia at 12 months. Pooled analysis of 5 randomized controlled trials demonstrating a statistically significant benefit of EIVOM-based strategies compared with control. Abbreviations: CI = confidence interval; EIVOM = ethanol infusion in the vein of Marshall; PVI = pulmonary vein isolation; RR = relative risk.

The EIVOM-based strategy demonstrated statistically significant superiority over PVI alone for the primary composite endpoint of freedom from any atrial arrhythmia at 12 months following a single ablation procedure. The pooled RR was 1.16 (95% CI 1.04–1.29; *I*^2^ = 0%, *P* < 0.001), with event rates of 74.5% in the EIVOM group vs. 62.7% in the control group (*Figure 3*). The absolute risk difference (ARD) was 10.3% (95% CI 5.1–15.5%; *P* = 0.0001), yielding NNT of 10 (95% CI 7–20). This clinically meaningful metric indicates that 10 patients would need to undergo the EIVOM-based ablation strategy rather than PVI alone to achieve one additional patient free from any arrhythmia recurrence at 12 months.

#### Freedom from atrial fibrillation

**Figure 4:**
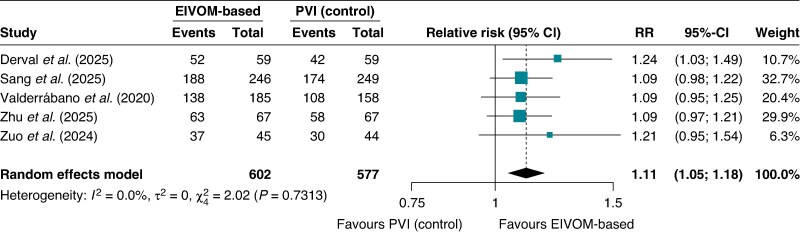
Forest plot of freedom from atrial fibrillation at 12 months. Pooled analysis indicating a statistically significant improvement in freedom from atrial fibrillation with EIVOM-based strategies compared with control. Abbreviations: CI = confidence interval; EIVOM = ethanol infusion in the vein of Marshall; PVI = pulmonary vein isolation; RR = relative risk.

When examining the specific endpoint of freedom from AF recurrence, the EIVOM group demonstrated statistically significant superiority compared with control (RR 1.11; 95% CI 1.05–1.18; *I*^2^ = 0%, *P* < 0.001), with success rates of 83.1% vs. 72.8%, respectively (*Figure 4*). The ARD of 8.3% (95% CI 3.6–13.0%; *P* = 0.0005) corresponded to an NNT of 13 (95% CI 8–28), indicating that 13 patients would require treatment with the EIVOM strategy to prevent one AF recurrence over 12 months.

#### Freedom from atrial tachycardia and atrial flutter

**Figure 5:**
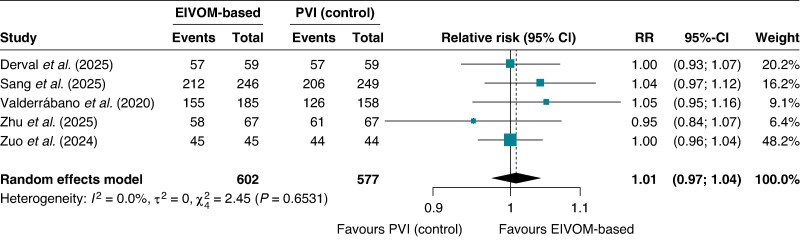
Forest plot of freedom from atrial tachycardia and atrial flutter at 12 months. Pooled analysis demonstrating comparable rates of freedom from organized atrial tachyarrhythmias (atrial tachycardia or atrial flutter) between EIVOM-based strategies and control groups. Abbreviations: CI = confidence interval; EIVOM = ethanol infusion in the vein of Marshall; PVI = pulmonary vein isolation; RR = relative risk.

In contrast to AF outcomes, there was no significant difference between treatment groups in freedom from AT or AFL (RR 1.01; 95% CI 0.97–1.04; I^2^ = 0%, *P* = 0.61), with event rates of 91.7% in the EIVOM group vs. 91.3% in controls (*Figure 5*). The ARD was 0.8% (95% CI −2.0 to 3.6%; *P* = 0.56).

#### Time-to-event analysis

**Figure 6:**
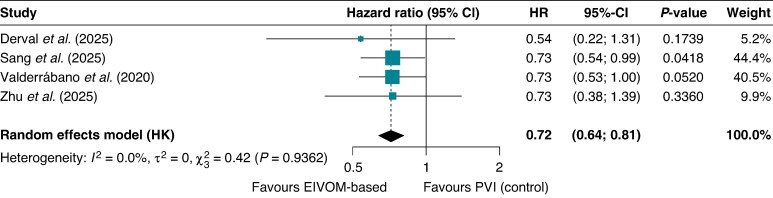
Forest plot of the time-to-event meta-analysis for any atrial arrhythmia. Pooled time-to-event analysis demonstrating a significant reduction in the hazard of any atrial arrhythmia recurrence at 12 months with EIVOM-based ablation strategies compared with PVI alone. Abbreviations: CI = confidence interval; EIVOM = ethanol infusion in the vein of Marshall; HR = hazard ratio; PVI = pulmonary vein isolation.

In the time-to-event meta-analysis, the EIVOM-based strategy was associated with a significantly lower rate of any atrial arrhythmia recurrence over 12 months compared with PVI alone (HR 0.72, 95% CI 0.64–0.81; *P* = 0.003; *I*^2^ = 0%). There was no evidence of statistical heterogeneity across studies, indicating consistent treatment effects among the included trials (*Figure 6*).

### Trial sequential analysis

**Figure 7:**
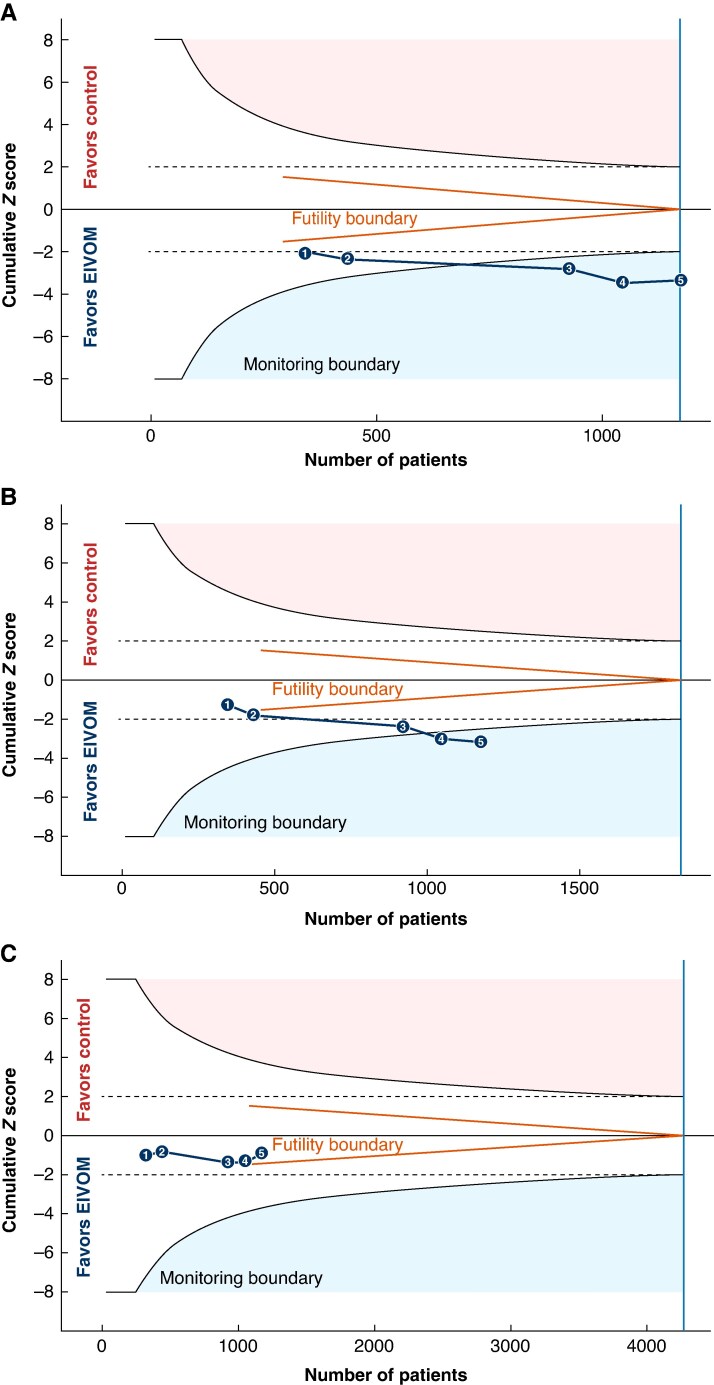
Trial sequential analysis of arrhythmia recurrence. Trial sequential analysis for (*A*) any arrhythmia recurrence, (*B*) atrial fibrillation recurrence, and (*C*) atrial tachycardia/flutter recurrence. The blue numbered line indicates cumulative Z-scores; curved lines indicate monitoring boundaries; and red wedges indicate futility boundaries. The vertical blue line represents the required information size. Numbered data points represent the cumulative addition of studies in chronological order: 1, Valderrábano et al; 2, Zuo et al; 3, Sang et al; 4, Derval et al; and 5, Zhu et al. Parameters: Two-sided α = 5%, Power = 80%, Relative Risk Reduction = 20%. Abbreviations: EIVOM = ethanol infusion of the vein of Marshall.

TSA confirmed the conclusive superiority of EIVOM-based strategies over PVI alone in reducing any arrhythmia recurrence, where the accrued sample size (*n* = 1179) surpassed the diversity-adjusted RIS (RIS = 1174; *D*^2^ = 6.4%). Analysis of AF recurrence demonstrated robust early efficacy, with the cumulative Z-score crossing the monitoring boundary for benefit prior to reaching the theoretical RIS (RIS = 1832; *D*^2^ = 0%). Conversely, the analysis for AT/AFL recurrence established futility, with the Z-score entering the futility boundary (RIS = 4270; *D*^2^ = 0%) (*Figure 7*).

### Procedural efficiency and acute outcomes

**Figure 8:**
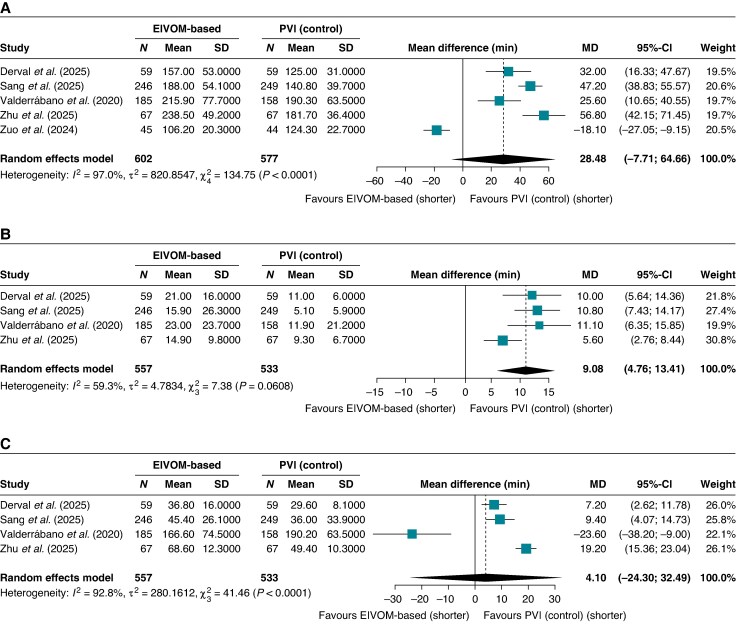
Forest plot of procedural efficiency metrics. Forest plots illustrating pooled mean differences for (*A*) total procedure time, (*B*) fluoroscopy time, and (*C*) total radiofrequency ablation time. Abbreviations: CI = confidence interval; EIVOM = ethanol infusion in the vein of Marshall; MD = mean difference; PVI = pulmonary vein isolation.

The EIVOM strategy was associated with a numerical increase in total procedural time (MD +28.48 min; 95% CI −7.71 to 64.66; *I*^2^ = 97%, *P* = 0.09), though this difference did not achieve statistical significance (*Figure 8A*). However, substantial heterogeneity was observed for this metric. Fluoroscopy exposure time was significantly longer in the EIVOM group, with moderate heterogeneity (MD +9.08 min; 95% CI 4.76–13.41; I^2^ = 59.3%, *P* = 0.007) (*Figure 8B*). Ablation time did not differ significantly between groups, with substantial heterogeneity (MD +4.1 min; 95% CI −24.30 to 32.49; *I*^2^ = 92.8%, *P* = 0.68) (*Figure 8C*).

### Mitral isthmus block achievement

**Figure 9:**
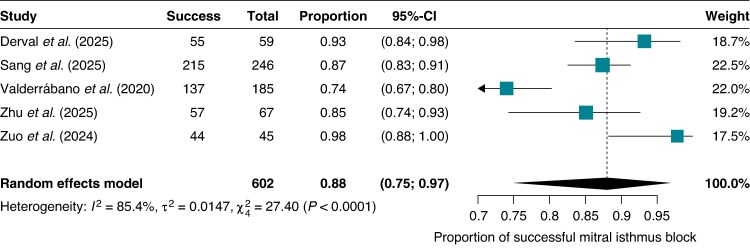
Acute mitral isthmus block achievement in the EIVOM arm. Forest plot illustrating the pooled proportion of successful bidirectional mitral isthmus block verified in patients treated with the EIVOM strategy. Abbreviations: CI = confidence interval; EIVOM = ethanol infusion in the vein of Marshall.

Successful acute MI bidirectional conduction block was achieved in 88% of EIVOM-treated patients (95% CI 0.75–0.97) across all five trials, with individual study success rates ranging from 74% (Valderrábano et al., 95% CI 0.67–0.80) to 98% (Zuo et al., 95% CI 0.88–1.00). Substantial heterogeneity was observed (*I*^2^ = 85.4%) (*Figure 9*).

Comparative analysis of MI block rates between EIVOM and control groups (*n* = 2) yielded inconclusive results (RR 1.29; 95% CI 0.33–5.09; *I*^2^ = 73.0%, *P* = 0.26) due to high imprecision, wide CIs, and substantial heterogeneity. Similarly, overall acute procedural success rates (RR 1.13; 95% CI 0.64–2.00; *I*^2^ = 91.4%, *P* = 0.59) demonstrated considerable uncertainty, precluding definitive conclusions regarding acute procedural endpoints (see Supplementary material online, *Figure S1*).

### Repeat ablation

**Figure 10:**
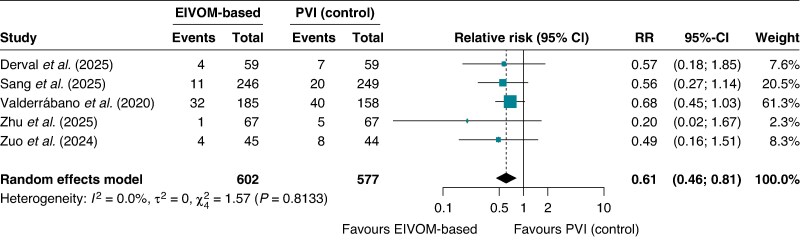
Forest plot of repeat ablation. This forest plot illustrates the relative risk of requiring a repeat ablation procedure. Abbreviations: CI = confidence interval; EIVOM = ethanol infusion in the vein of Marshall; PVI = pulmonary vein isolation; RR = relative risk.

The EIVOM strategy conferred a significant reduction in the need for subsequent ablation procedures. The pooled repeat ablation rate was 7.13% in the EIVOM group compared with 13.50% in controls, yielding a RR of 0.61 (95% CI 0.46–0.81; *I*^2^ = 0%, *P* = 0.009). Heterogeneity was minimal, indicating a consistent reduction in repeat procedure burden (*Figure 10*). The EIVOM strategy yielded an ARD for repeat ablation of 5.0% (95% CI: 1.9% to 8.1%; *P* = 0.002), corresponding to an NNT of 20 (95% CI 13–53) to prevent one re-intervention.

### Safety outcomes

#### Overall complication profile

**Figure 11:**
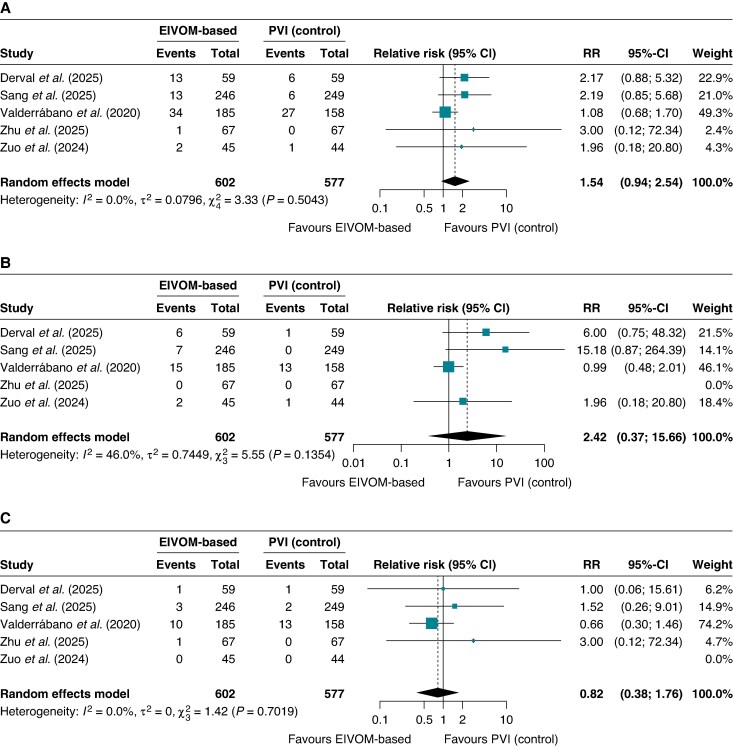
Safety endpoints. Forest plots illustrating pooled relative risks for (*A*) the overall complication profile, (*B*) pericardial effusion or pericarditis, and (*C*) major adverse events. Abbreviations: CI = confidence interval; EIVOM = ethanol infusion in the vein of Marshall; PVI = pulmonary vein isolation; RR = relative risk.

Any complication occurred in 63 of 602 patients (10.5%) in the EIVOM group compared with 40 of 577 patients (6.9%) in the control group. While this represented a numerical trend towards increased periprocedural risk with EIVOM, the RR did not reach statistical significance (RR 1.54; 95% CI 0.94–2.54; I^2^ = 0%, *P* = 0.07) (*Figure 11A*). Heterogeneity among the included trials was absent.

#### Pericardial complications

Pericardial effusion or pericarditis occurred more frequently in the EIVOM group, affecting 30 patients (5.0%) vs. 14 control patients (2.6%). The RR estimate suggested an increased risk (RR 2.42; 95% CI 0.37–15.66; *I*^2^ = 46.0%, *P* = 0.23), though the extremely wide CI spanning more than 40-fold reflects the relatively low event rate and moderate heterogeneity between studies (*Figure 11B*). Cardiac tamponade, the most serious pericardial sequela requiring urgent intervention, was rare in both groups: 3 events (0.5%) in EIVOM-treated patients vs. 1 event (0.2%) in controls (RR 2.33; 95% CI 0.34–15.81; *P* = 0.39) (see Supplementary material online, *Figure S2*). The absolute risk increase, if any, remains very small (0.3%), and the rarity of this outcome limits statistical power to detect meaningful differences.

#### Major adverse events

Major complications occurred in 15 EIVOM-treated patients (2.49%) vs. 16 control patients (2.77%). The RR was 0.82 (95% CI 0.38–1.76; I^2^ = 0%, *P* = 0.47), with minimal heterogeneity (*Figure 11C*). The ARD was 0.25% (95% CI −1.14 to 1.64%; *P* = 0.73).

#### Other complications

No phrenic nerve injuries were reported in either treatment group across all 5 trials, despite extensive ablation along the lateral mitral annulus and right atrial cavotricuspid isthmus.

#### Sensitivity analyses

Sensitivity analyses confirmed the robustness of all findings (see Supplementary material online, *Figure S9*–*S19*): sequential omission of individual studies yielded a pooled RR that remained stable with minimal changes, and no single trial materially altered the magnitude or statistical significance of results. These findings demonstrate that the overall treatment effect is not driven by any one study and is consistently reproducible across the available evidence.

## Discussion

This systematic review and meta-analysis, comprising 5 RCTs and 1179 patients, provides the most rigorous evidence to date regarding the efficacy and safety of EIVOM-based strategies compared with PVI for PeAF. Our findings demonstrate that the EIVOM strategy yields a robust and clinically meaningful improvement in freedom from any atrial arrhythmia and freedom from AF at 12 months, with an NNT of 10 and 13, respectively. Importantly, time-to-event analysis demonstrated a sustained 28% relative reduction in recurrence hazard throughout follow-up, indicating that the benefit reflects a consistent attenuation of arrhythmia risk over time rather than a divergence at a single fixed time point. Additionally, the EIVOM-based strategy significantly reduces the burden of repeat ablation procedures. While EIVOM is associated with increased fluoroscopy duration and a modest increase in minor pericardial complications, it does not increase the risk of major adverse events. These data suggest that EIVOM effectively targets the complex, extra-pulmonary substrate of PeAF, offering a mechanistic advantage over conventional endocardial ablation alone. However, while our analysis demonstrates superior arrhythmia-free survival with the intervention, it is critical to recognize that EIVOM was rarely performed as an isolated adjunct across the included trials. Rather, EIVOM was frequently integrated into a comprehensive ‘Marshall Plan’ or broader substrate modification strategy, which variably included additional linear lesions such as MI and LA roof lines. Consequently, the observed reduction in atrial arrhythmia recurrence cannot be exclusively attributed to the chemical ablation of the Marshall bundle alone. Instead, EIVOM likely facilitates the achievement of durable MI block, a frequent site of recurrence in PeAF and redo ablation procedures.^15^ Therefore, our findings support the clinical efficacy of an EIVOM-facilitated ablation strategy, rather than evaluating ethanol infusion as a solitary, isolated variable.

### Mechanistic validation and clinical context

These findings provide another perspective on the ongoing debate regarding the optimal ablation strategy for PeAF. Historically, the landmark STAR AF II trial demonstrated that the empiric addition of linear lesions (roof and mitral lines) to PVI provided no incremental benefit over PVI alone, with freedom from recurrent AF rates plateauing at approximately 59%, at 18 months.^32^ This failure has often been attributed to the inability of standard RF energy to achieve durable transmurality across the varying thickness of the MI.^33,34^ In stark contrast, our meta-analysis demonstrates that when substrate modification is anatomically specific (targeting the Marshall bundle) and utilizes a mechanism-based modality (ethanol infusion), substantial clinical benefit is realized (83.1% freedom from AF vs. 59% in STAR AF II).^32^ This distinction is important: the failure of previous linear strategies likely reflected the limitations of the energy source rather than the invalidity of the anatomical target. Multiple studies have demonstrated that achieving MI block through conventional RF ablation is hampered by the ‘heat-sink’ effect of epicardial vessels that remove thermal energy through convective cooling, preventing transmural lesion formation.^33–36^ Additionally, the presence of myocardial sleeves around the CS and the VoM can act as epicardial bridges of conduction, bypassing endocardial ablation lines and necessitating epicardial ablation in 50–70% of cases.^16,37^ Wong et al. demonstrated that most MI reconnections occur at the annular end of the ablation line, precisely where these epicardial vascular structures exert their maximal heat-sink effect.^33^

By overcoming the epicardial heat-sink effect through chemical ablation to facilitate transmural lesions independent of blood flow or catheter contact, EIVOM validates that precise targeting of extra-pulmonary substrates, rather than non-selective empirical ablation, is required to improve PeAF ablation success.^15,38^ The VoM, as a remnant of the embryonic left superior vena cava containing the Marshall bundle, represents a mechanistically defined anatomical substrate rather than an arbitrary linear lesion location.^12,39^ The Marshall bundle exhibits rapid activation and complex fractionated electrograms during persistent AF, suggesting a role in driving or perpetuating AF beyond its function as an epicardial conduction bridge.^13,40^ EIVOM eliminates this substrate comprehensively through direct tissue necrosis while simultaneously denervating surrounding autonomic ganglia, addressing both the anatomical and neurohormonal mechanisms of AF perpetuation.^20^ This distinguishes EIVOM from previous empirical linear ablation approaches tested in STAR AF II, which targeted arbitrary anatomical locations (roof line, MI line) without addressing the underlying epicardial substrate or heat-sink challenges inherent to those regions.^32^ The consistency of EIVOM benefit across all 5 RCTs in the present meta-analysis (*I*^2^ = 0%) contrasts with the inconsistent results of CFAE-guided ablation and empirical linear lesions across multiple trials.^3^

A nuanced finding of this meta-analysis is the differential impact of EIVOM on AF vs. organized ATs (AT/AFL). The clinical benefit was driven almost exclusively by enhanced suppression of AF (RR 1.11 for freedom from AF), whereas freedom from AT/AFL was identical between groups (RR 1.01). This observation is clinically important. Extensive atrial ablation employing linear lesions has historically been criticized for potentially creating pro-arrhythmic gaps that promote iatrogenic flutter.^41,42^ Multiple studies have demonstrated that incomplete MI lines substantially increase the risk of developing perimitral flutter, with gaps serving as critical re-entrant circuit isthmuses.^41,43,44^ Knecht et al. demonstrated that a gap in the MI line is a prerequisite for post-procedural macroreentrant perimitral flutter, occurring in 76% of patients with incomplete lines compared with 35% of those achieving bidirectional block.^43^

The fact that EIVOM, which facilitates the creation of a mitral line, did not increase the incidence of organized tachycardias suggests that the lesions created are more likely to be transmural and durable than those achieved by RF alone. By solidifying the mitral block through chemical ablation that is independent of heat-sink effects, catheter contact force, and epicardial fat thickness, EIVOM likely prevents the macro-reentrant circuits that typically arise from incomplete endocardial ablation.^15,44^ This is corroborated by the present meta-analysis, demonstrating that MI block was achieved in 87% of EIVOM-treated patients compared with only 74% in STAR AF II using RF alone.^32^ The NNT of 10 for freedom from any arrhythmia highlights a high return on procedural investment, positioning EIVOM as one of the most effective adjunctive strategies for PeAF.

Prior meta-analyses have been limited by the inclusion of non-randomized observational cohorts, which introduces substantial selection bias, often favoring EIVOM in healthier patients or confounding results with varying operator skill levels.^23,24,45^ A recent meta-analysis by Itaya et al. included 4 RCTs and 16 observational studies, demonstrating that restriction to RCTs yielded a more conservative effect estimate (OR 0.58) compared with the combined observational-RCT estimate (OR 0.51), highlighting the risk of overestimation from non-randomized data.^24^ Similarly, a systematic review by Li et al., including both RCTs and observational studies, reported an RR of 1.28 for long-term freedom from AF/AT but acknowledged substantial heterogeneity (*I*^2^ = 68.9%) attributable to study design differences and confounding.^45^ By restricting our analysis strictly to RCTs with appropriate blinding of outcome assessors, we provide a more unadulterated estimate of the treatment effect with minimal heterogeneity. A significant strength of this study is the application of TSA.^31^ TSA suggests that the RIS has been met to demonstrate a significant reduction in arrhythmia-recurrence associated with EIVOM-based ablation at 12 months. This approach supports the robustness of the current mid-term data; however, these results must be interpreted within the context of the available 12-month follow-up window. Longer-term, time-to-event analyses are still required to confirm the extended durability of this intervention. TSA also delineated a mechanistic distinction between arrhythmia subtypes. While the reduction of AF crossed the monitoring boundary, the analysis for AT/AFL indicated futility. This suggests that the clinical benefit of an EIVOM-based strategy is driven primarily by the suppression of AF rather than organized ATs.

### Procedural efficiency and practical implementation

EIVOM-based strategies were associated with a significant increase in fluoroscopy duration (+9.08 min; *P* = 0.007) but did not significantly prolong total procedure time (*P* = 0.09) or RF ablation duration (*P* = 0.68). Notably, total procedure time and RF ablation duration demonstrated substantial statistical heterogeneity (*I*^2^ = 97% and 92.8%, respectively). Given the inclusion of only five trials, meta-regression analyses were underpowered to explore potential study-level effect modifiers. Accordingly, these procedural outcomes should be interpreted as descriptive estimates, likely reflecting differences in centre-specific workflows and operator practice patterns. This dissociation between times suggests that the incremental fluoroscopy requirements reflect the technical demands of VoM cannulation and ethanol visualization rather than a protracted procedural workflow. Evidence indicates a steep learning curve, with fluoroscopy utilization decreasing significantly as centres transition from the initial adoption phase to steady state practice.^46^ Indeed, while a contemporary single-centre series reported VoM identification and cannulation success rates of 98.1% and 92.6%, demonstrating that high success rates are achievable with dedicated experience,^47^ broader trial data reflect persistent anatomical hurdles. For instance, the PROMPT-AF trial reported that 15% of patients assigned to EIVOM could not undergo the procedure, primarily due to an inability to visualize the VoM using venography (12.2%) or failed cannulation (3.8%).^19^ This corroborates a large-scale observational study by Kamakura et al., of 713 consecutive patients, which demonstrated successful VoM ethanol infusion in 88.9% of cases; failures were predominantly driven by non-identification or non-cannulation, challenges that can be partially mitigated by utilizing anatomical landmarks such as the Valve of Vieussens.^48^ Furthermore, strategies such as a ‘VoM-first’ sequence (EIVOM prior to PVI) may optimize outcomes by preserving VoM ostial patency and enhancing visualization, potentially resulting in shorter procedural times and higher first-pass block rates compared to RF-first approaches.^49^ Nonetheless, contemporary evidence validating VoM strategies is predominantly derived from highly experienced, high-volume tertiary centres, representing an inherent limitation of the present meta-analysis. Consistent visualization and precise cannulation of the VoM demand a profound appreciation of highly variable coronary venous anatomy. Consequently, the high technical success rates reported in contemporary trials inherently reflect the specialized acumen of these expert operators. Such robust outcomes cannot be reflexively anticipated in lower-volume settings, underscoring that the safety and efficacy of EIVOM-based strategies remain inextricably linked to operator experience, thereby constraining their broad generalizability and routine clinical adoption.

In the present analysis of *de novo* cases, EIVOM-treated patients demonstrated a significantly lower requirement for repeat ablation (RR 0.61; 95% CI 0.46–0.81). However, the role of EIVOM during the redo procedure itself remains a subject of active debate. The 2024 EHRA survey by Conti et al., reported that EIVOM is infrequently adopted as an empirical ablation target during repeat procedures (8.6%), with posterior wall isolation representing the more commonly pursued empirical strategy (20.7%).^50^ Observational data from Kneizeh et al., suggest that VoM elimination combined with complimentary linear lesions, may serve as a potential salvage strategy in redo cases with durable PVI but recurrent perimitral flutter.^51^ Conversely, a retrospective study by Hsia et al. found no incremental benefit when EIVOM was added to redo procedures with prior substrate modification.^52^ These conflicting data, coupled with the low adoption rate reported by Conti et al., suggest that while EIVOM is an effective tool for index procedures to prevent recurrence, its application in the redo setting is currently a niche strategy.^50^ It should likely be reserved for patients where the VoM is identified as a specific localized driver or where durable MI block has previously failed.

### Safety profile and risk–benefit analysis

Heterogeneous reporting of complications across trials and the relatively small event numbers create statistical imprecision for rare adverse events. Any-complication incidence was numerically higher in the EIVOM group (10.5% vs. 6.9%; *P* = 0.07), reflecting the inherent trade-off of more extensive ablation strategies and broader anatomical modification, which may provoke a more pronounced pericardial inflammatory response.^32,53,54^ Notably, major adverse events occurred at similar rates between groups (2.5% vs. 2.8%; *P* = 0.47), indicating that the observed increase in overall complications is not driven by severe outcomes.

Pericardial effusion (5.0% vs. 2.6%; RR 2.42) represented the primary driver of increased any-complication incidence. The observed pericardial effusion rate of 5.0% with EIVOM falls within the range reported for contemporary AF ablation procedures in large registry studies (1.2–14.2%).^55,56^ In this analysis, most EIVOM-associated effusions were mild to moderate, managed conservatively with observation, pericardiocentesis, or brief hospitalization. Comparing the EIVOM-associated cardiac tamponade rate (0.5%) with published rates in conventional PVI procedures reveals no excess absolute risk.^57,58^ Recent data from an EHRA survey by Bordignon et al. further emphasize that while overall per-patient complication rates in contemporary AF ablation are relatively low, high-severity events are persistently encountered by European electrophysiologists.^59^ Specifically, 89% of surveyed operators report having experienced a case of cardiac tamponade.^59^ The persistent reality of these potentially lethal complications, even among experienced operators, reinforces the necessity of carefully weighing incremental efficacy against procedural complexity and risk when considering intensified, EIVOM-based substrate modification strategies.

### Limitations

Our study has several important limitations. First, the small number of included studies (*n* = 5) limited the statistical power for detecting publication bias (Egger’s regression requires ≥10 studies) or performing a robust multivariable meta-regression to adjust for potential confounders such as LA volume or AF duration. Although univariate meta-regression was performed for baseline characteristics, no significant associations were identified for any endpoint (see Supplementary material online, *Table S3*). Secondly, reliance on aggregate rather than individual patient data necessitated the digitization of Kaplan–Meier curves for Zhu et al., introducing measurement imprecision, although this method is validated.^21,29^ Third, there was substantial procedural heterogeneity within the ‘Marshall Plan’ strategy. Although all intervention arms incorporated EIVOM, the extent and configuration of adjunctive lesion sets varied between studies, including differences in dome line use and definitions of MI line endpoints.^18^ As the comparator in all trials was PVI alone, the addition of these lesion sets in the EIVOM arms may have contributed to the observed treatment effect and cannot be fully disentangled. While statistical heterogeneity for efficacy outcomes was low, this underlying clinical heterogeneity suggests that EIVOM-based ablation represents a spectrum of more extensive ablation strategies rather than a single standardized technique. Fourth, the inherent open-label design of procedural trials introduces potential performance bias. Knowledge of treatment allocation may have influenced operator persistence in achieving bidirectional block in the control arm, although the use of objective Holter-based endpoints mitigates detection bias. However, rhythm-monitoring protocols were heterogeneous across the five RCTs (*Table 1*). While all trials mandated scheduled 12-lead ECGs and at least intermittent ambulatory monitoring, intensity ranged from weekly transtelephonic or patch-based recordings (Marshall-Plan, PROMPT-AF) to 30-day continuous external monitoring at 6 and 12 months (VENUS) or periodic 24-h to 7-day Holter protocols (Zhu, Zuo).^18–22^ Notably, systematic implantation of loop recorders was not undertaken in any included study. While definitions of recurrence were broadly aligned, non-uniform monitoring intensity may have influenced the detection of asymptomatic recurrences and influenced absolute event rates. This stands in contrast to the 2024 EHRA–HRS–APHRS–LAHRS consensus statement, which recommends implantable loop recorders or similarly intensive monitoring when feasible to provide comprehensive post-ablation surveillance.^1^ The absence of such systematic device-based monitoring represents an inherent limitation that potentially results in the under-detection of late arrhythmia recurrences. Moreover, a notable limitation of the present analysis is the observed geographic variation in treatment effect. A prespecified subgroup analysis (see Supplementary material online, *Figure S6*) demonstrated a highly significant interaction (χ^2^ = 19.93; *P* < 0.0001), with a larger treatment effect in non-China studies (RR 1.30; 95% CI 1.23–1.38; *I*^2^ = 0.0%) compared with China-based studies (RR 1.12; 95% CI 0.96–1.29; *I*^2^ = 0.0%). Although statistical heterogeneity within subgroups was absent, the magnitude and direction of this interaction raise the possibility of regional effect modification. These differences may reflect variation in patient selection, atrial substrate characteristics, procedural standardization, adjunctive ablation strategies, or operator experience. Consequently, while the overall pooled estimate favours EIVOM-based therapy, the significant geographic interaction tempers the certainty of universal generalisability and should be interpreted as hypothesis-generating pending confirmation in standardized multinational cohorts.

Finally, the follow-up duration was uniformly capped at 12 months. Given the progressive nature of atrial myopathy, the long-term durability of the autonomic denervation and chemical ablation effects achieved by EIVOM remains unknown. Additionally, as this meta-analysis was restricted to thermal ablation, these findings cannot be extrapolated to emerging modalities such as pulsed field ablation (PFA), where the differential effect of EIVOM on lesion durability and safety has yet to be established in randomized settings.^60,61^

### Future directions

Several critical research gaps warrant investigation. First, long-term durability beyond 12 months remains unknown. While preliminary observational data suggest sustained efficacy at 24–36 months, prospective long-term substudies nested within future RCTs are needed to establish whether efficacy gains persist or are subject to late recurrence.^45,62^ Moreover, randomized comparative effectiveness trials directly contrasting EIVOM against emerging alternatives (e.g. head-to-head comparisons with PFA) are lacking.^61^ Such trials, such as the anticipated VEMAPULSE trial (NCT06383975), would clarify whether EIVOM represents the optimal substrate-based approach or whether alternative techniques offer superior efficacy-safety trade-offs.^1^

Furthermore, health economic analyses incorporating procedural costs, hospitalization expenses, outpatient follow-up burden, and quality-of-life measures would contextualize the clinical efficacy findings within the broader health system frameworks. Evaluating the performance of EIVOM-based strategies in lower-volume centres would provide an informative insight into their generalizability and feasibility in real-world, non-tertiary settings. While the NNT of 10 represents substantial clinical benefit, the absolute cost-effectiveness of EIVOM-based ablation relative to alternative strategies or ongoing pharmacotherapy remains unknown.

## Conclusion

In patients with PeAF, the integration of EIVOM-based strategies into comprehensive catheter ablation significantly improves 12-month arrhythmia-free survival and reduces the necessity for repeat ablation when compared to non-ethanol PVI-based approaches. However, these mid-term benefits must be interpreted cautiously. The incremental efficacy observed likely reflects a broader ‘Marshall Plan’ substrate modification approach rather than the isolated effect of chemical ablation alone. Furthermore, the technique remains technically demanding, with contemporary evidence predominantly derived from highly experienced, high-volume tertiary centres, thereby limiting its immediate generalizability. While major complications were not significantly increased in the trial setting, the potential for severe procedural morbidity persists. Consequently, rather than unqualified routine adoption, EIVOM-based approaches should be considered a mechanistically targeted adjunct in experienced centres, particularly when a durable MI block is a predefined procedural objective. Future investigations must prioritize long-term follow-up beyond 12 months, systematic device-based rhythm monitoring, and comparative effectiveness trials against emerging non-thermal modalities.

## Supplementary Material

euag069_Supplementary_Data

## Data Availability

The data underlying this article are available in the article and in its online Supplementary material.
