# Supplementary material for: Ethanol infusion of the vein of marshall-based strategies for persistent atrial fibrillation: a systematic review and meta-analysis of randomized trials
Source: Europace. 2026 Apr 2;28(4):euag069. doi: 10.1093/europace/euag069 (PMC13122365; doi:10.1093/europace/euag069)
Supplement: euag069_Supplementary_Data [file euag069_supplementary_data.docx]

**Supplementary material**

**Supplementary Table S1: Search Strategy for PubMed, Web of Science, and MEDLINE (Ovid)**

| **Search Terms** |
| --- |
| **PubMed Search Strategy:** |
| ("Atrial Fibrillation"[Mesh] OR "atrial fibrillation"[tiab] OR "persistent atrial fibrillation"[tiab] OR "chronic atrial fibrillation"[tiab] OR "AF"[tiab]) AND ("Marshall"[tiab] OR "Vein of Marshall"[tiab] OR "Marshall vein"[tiab] OR "Marshall bundle"[tiab] OR "Oblique Vein of Marshall"[tiab] OR "VOM"[tiab]) AND ("Ethanol"[Mesh] OR "Alcohols"[Mesh] OR "ethanol"[tiab] OR "alcohol"[tiab] OR "infusion"[tiab] OR "injection"[tiab] OR "ablation"[tiab] OR "Catheter Ablation"[Mesh] OR "Ablation Techniques"[Mesh] OR "catheter ablation"[tiab] OR "radiofrequency ablation"[tiab] OR "cryoablation"[tiab]) |
|  |
| **Web of Science Search Strategy:** |
| ("atrial fibrillation" OR "persistent atrial fibrillation" OR "chronic atrial fibrillation" OR "AF") AND ("Vein of Marshall" OR "Marshall vein" OR "Marshall bundle" OR "Oblique Vein of Marshall" OR "Marshall" OR "VOM") AND ("ethanol" OR "alcohol" OR "ethanol infusion" OR "ethanol ablation" OR "infusion" OR "ablation" OR "catheter ablation" OR "radiofrequency ablation") |
|  |
| **MEDLINE (Ovid) Search Strategy:** The search combined three concept groups limited to human subjects: Group 1 (Atrial Fibrillation): exp Atrial Fibrillation/ OR atrial fibrillation.tw. OR persistent atrial fibrillation.tw. OR AF.tw.; Group 2 (Vein of Marshall): Marshall.tw. OR Vein of Marshall.tw. OR Marshall bundle.tw. OR VOM.tw.; Group 3 (Ablation Techniques): exp Ethanol/ OR exp Catheter Ablation/ OR ethanol.tw. OR ablation.tw. OR catheter ablation.tw. |

## **Calculation Method**

Both NNT and NNH were derived from the pooled absolute risk difference (ARD) obtained through random-effects meta-analysis using the Mantel-Haenszel method. The ARD was calculated as:

ARD=P_EIVOM_−P_Control_

Where P_EIVOM_ and P_Control_ represent the pooled event proportions in the EIVOM and control groups, respectively.

## **NNT Calculation (Beneficial Outcomes)**

For efficacy endpoints where EIVOM resulted in improved outcomes (higher event rates representing success), NNT was calculated as the reciprocal of the absolute value of the pooled ARD:

NNT=$\frac{1}{\mid ARD\mid}$ (when ARD > 0)

**Supplementary Table S2: Baseline Characteristics of Included Studies**

| **Characteristic** | **Study^18-22^** | **EIVOM** | **Control** |
| --- | --- | --- | --- |
| **Total Patients (N)** | Derval et al. | 59 | 59 |
|  | Sang et al. | 246 | 249 |
|  | Valderrábano et al. | 185 | 158 |
|  | Zhu et al. | 67 | 67 |
|  | Zuo et al. | 45 | 44 |
|  | **Overall Total** | **602** | **577** |
| **Age (years, Mean ± SD)** | Derval et al. | 66 ± 8 | 65 ± 8 |
|  | Sang et al. | 61.3 ± 9.9 | 61 ± 9.5 |
|  | Valderrábano et al. | 66.6 ± 9.6 | 66.4 ± 9.9 |
|  | Zhu et al. | 60.8 ± 7.7 | 59.9 ± 9.9 |
|  | Zuo et al. | 63 ± 6.3 | 62.8 ± 6.2 |
|  | **Overall Mean Age** | **63.5** | **62.9** |
| **Female (n, %)** | Derval et al. | 12 (20%) | 9 (15%) |
|  | Sang et al. | 66 (26.8%) | 68 (27.3%) |
|  | Valderrábano et al. | 48 (26%) | 34 (22%) |
|  | Zhu et al. | 11 (16.4%) | 19 (28.4%) |
|  | Zuo et al. | 20 (44.4%) | 18 (40.9%) |
|  | **Overall Female** | **157 (26.0%)** | **148 (25.6%)** |
| **BMI (kg/m², Mean ± SD)**^†^ | Derval et al. | NR | NR |
|  | Sang et al. | NR | NR |
|  | Valderrábano et al. | 31.2 ± 6.6 | 31.9 ± 6.5 |
|  | Zhu et al. | 26.8 ± 3.3 | 26.8 ± 3.0 |
|  | Zuo et al. | NR | NR |
|  | **Overall Mean BMI** | **30.0** | **30.4** |
| **Hypertension, n (%)** | Derval et al. | 36 (60%) | 25 (42%) |
|  | Sang et al. | 136 (55.3%) | 130 (52.2%) |
|  | Valderrábano et al. | 144 (77%) | 104 (66%) |
|  | Zhu et al. | 35 (52.2%) | 37 (52.2%) |
|  | Zuo et al. | 25 (55.6%) | 26 (59.1%) |
|  | **Overall Hypertension** | **376 (62.4%)** | **322 (55.7%)** |
| **Diabetes mellitus, n (%)** | Derval et al. | 9 (15%) | 3 (5%) |
|  | Sang et al. | 32 (13%) | 27 (10.8%) |
|  | Valderrábano et al. | 52 (28%) | 31 (20%) |
|  | Zhu et al. | 6 (9.0%) | 10 (14.9%) |
|  | Zuo et al. | 6 (13.3%) | 6 (13.6%) |
|  | **Overall Diabetes** | **105 (17.4%)** | **77 (13.3%)** |
| **Heart failure, n (%)**^‡^ | Derval et al. | NR | NR |
|  | Sang et al. | 22 (8.9%) | 31 (12.4%) |
|  | Valderrábano et al. | 48 (26%) | 42 (27%) |
|  | Zuo et al. | NR | NR |
|  | Zuo et al. | 5 (11.1%) | 4 (9.1%) |
|  | **Overall, Heart Failure** | **75 (15.8%)** | **77 (17.1%)** |
| **Stroke/TIA, n (%)** | Derval et al. | 5 (8.3%) | 2 (3.3%) |
|  | Sang et al. | 14 (5.7%) | 17 (6.8%) |
|  | Valderrábano et al. | 19 (10%) | 19 (12%) |
|  | Zhu et al. | 2 (3.0%) | 4 (6.0%) |
|  | Zuo et al. | 1 (2.2%) | 1 (2.3%) |
|  | **Overall Stroke/TIA** | **41 (6.8%)** | **43 (7.4%)** |
| **CHA₂DS₂-VASc Score**^§^ | Derval et al. | 2 ± 1 | 2 ± 1 |
|  | Sang et al. | 1 [1-2] | 1 [0-2] |
|  | Valderrábano et al. | 2.9 ± 1.6 | 2.6 ± 1.6 |
|  | Zhu et al. | 1.3 ± 1.0 | 1.6 ± 1.3 |
|  | Zuo et al. | 1.8 ± 1.1 | 1.8 ± 0.9 |
|  | **Overall Mean Score** | **2.3** | **2.2** |
| **LA Size (mm, Mean ± SD)^¶^** | Derval et al. | 187±53 (Vol) | 192±53 (Vol) |
|  | Sang et al. | 42.8±6.1 (Diam) | 42.8±4.5 (Diam) |
|  | Valderrábano et al. | 44.8±7.9 (Diam) | 47.0±7.5 (Diam) |
|  | Zhu et al. | 45.8±2.4 (Diam) | 46.3±2.9 (Diam) |
|  | Zuo et al. | 42.3±3.1 (Diam) | 43.3±3.1 (Diam) |
|  | **Overall Mean Diameter** | **43.8** | **44.6** |
| **LVEF (%, Mean ± SD)** | Derval et al. | 51 ± 12 | 56 ± 10 |
|  | Sang et al. | 61.3 ± 7.6 | 61.1 ± 8 |
|  | Valderrábano et al. | 52.1 ± 10.1 | 53.4 ± 9.4 |
|  | Zhu et al. | 61.0 ± 5.2 | 59.2 ± 6.3 |
|  | Zuo et al. | 57 ± 5.2 | 58.1 ± 5.1 |
|  | **Overall Mean LVEF** | **57.1** | **58.0** |
| **AF Duration (months)**^#^ | Derval et al. | 9 ± 19 | 7 ± 6 |
|  | Sang et al. | 12 [5-24] | 12 [4-24] |
|  | Valderrábano et al. | NR | NR |
|  | Zhu et al. | 18 [12-36] | 18 [18-24] |
|  | Zuo et al. | 26.4 ± 14.4 | 28.8 ± 16.8 |
|  | **Overall Mean Duration** | **16.53** | **16.31** |

*Data presented as Mean ± SD, Median [IQR], or n (%). NR = Not Reported. Diam = Diameter. LA = Left Atrial. LVEF = Left Ventricular Ejection Fraction. Vol = Volume.*

- ^†^***BMI:*** *Pooled calculation only includes studies reporting this characteristic (Valderrábano et al., Zhu et al.; EIVOM N=252, Control N=225).*
- ^‡^***Heart failure:*** *Pooled calculation only includes studies reporting this (Sang et al., Valderrábano et al., Zuo et al.; EIVOM N=476, Control N=451).*
- ^§^***CHA₂DS₂-VASc:*** *Pooled calculation only includes studies reporting Mean±SD (Derval et al., Valderrábano et al., Zhu et al., Zuo et al.; EIVOM N=357, Control N=329).*
- **^¶^*LA Size:*** *The "Overall (Diam)" row represents the pooled mean diameter from the four studies that reported it.*
- ^#^***AF Duration:*** *Pooled calculation only includes studies reporting Mean±SD (Derval et al., Zuo et al.; EIVOM N=105, Control N=104).*

**
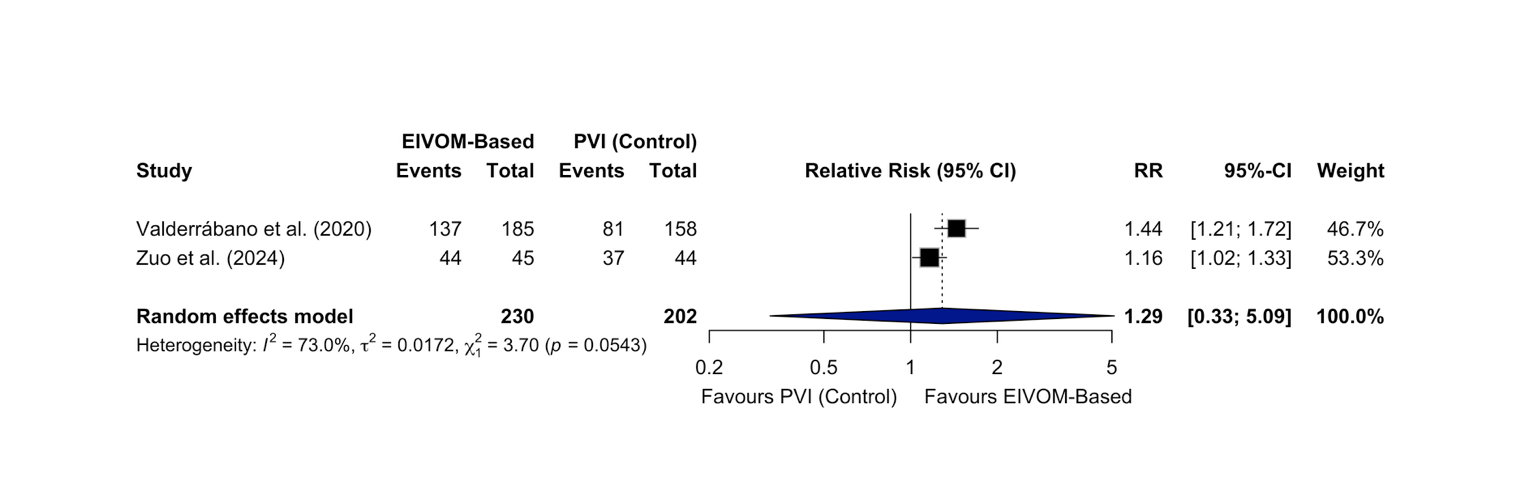
Supplementary Figure S1:** Comparative analysis of MI block rates between EIVOM and control groups

**Supplementary Figure S2:** Cardiac tamponade between the EIVOM and control groups.


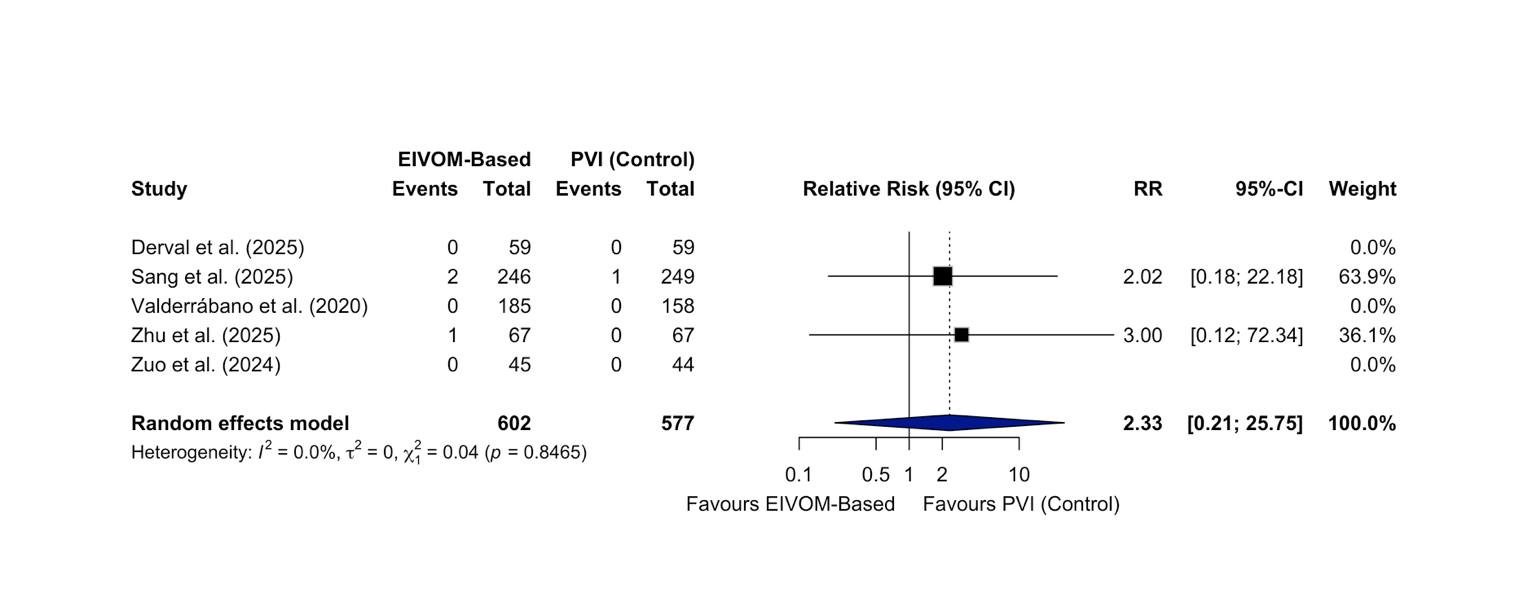


**Subgroup Analyses**

**Single-Center Versus Multicenter Trials**

Subgroup analysis stratified by trial design (single center versus multicenter) demonstrated consistent treatment effects across study settings. For freedom from any arrhythmia, both single-center trials (RR 1.16; 95% CI 0.86–1.58; I² = 31.3%) and multicenter trials (RR 1.16; 95% CI 0.59–2.27; I² = 0.0%) yielded identical point estimates with no significant subgroup difference (χ² = 0.00; P = 0.97), confirming that EIVOM benefit is not restricted to specialized high-volume centers (**Supplementary Figure S3**).

Freedom from AF was similarly consistent across settings (single-center RR 1.14, 95% CI 0.95–1.37; multicenter RR 1.09, 95% CI 1.08–1.11; χ² = 0.97; P = 0.32), supporting generalizability across diverse operator experiences and institutional workflows (**Supplementary Figure S4)**. For freedom from AT/AFL, both subgroups demonstrated no treatment effect (single-center RR 1.00, 95% CI 0.95–1.04; multicenter RR 1.04, 95% CI 0.99–1.10; χ² = 20.06; P < 0.0001), confirming that EIVOM does not increase organized arrhythmia risk regardless of trial setting (**Supplementary figure S5**). These findings support the external validity of EIVOM-based ablation strategies for implementation across diverse clinical settings.


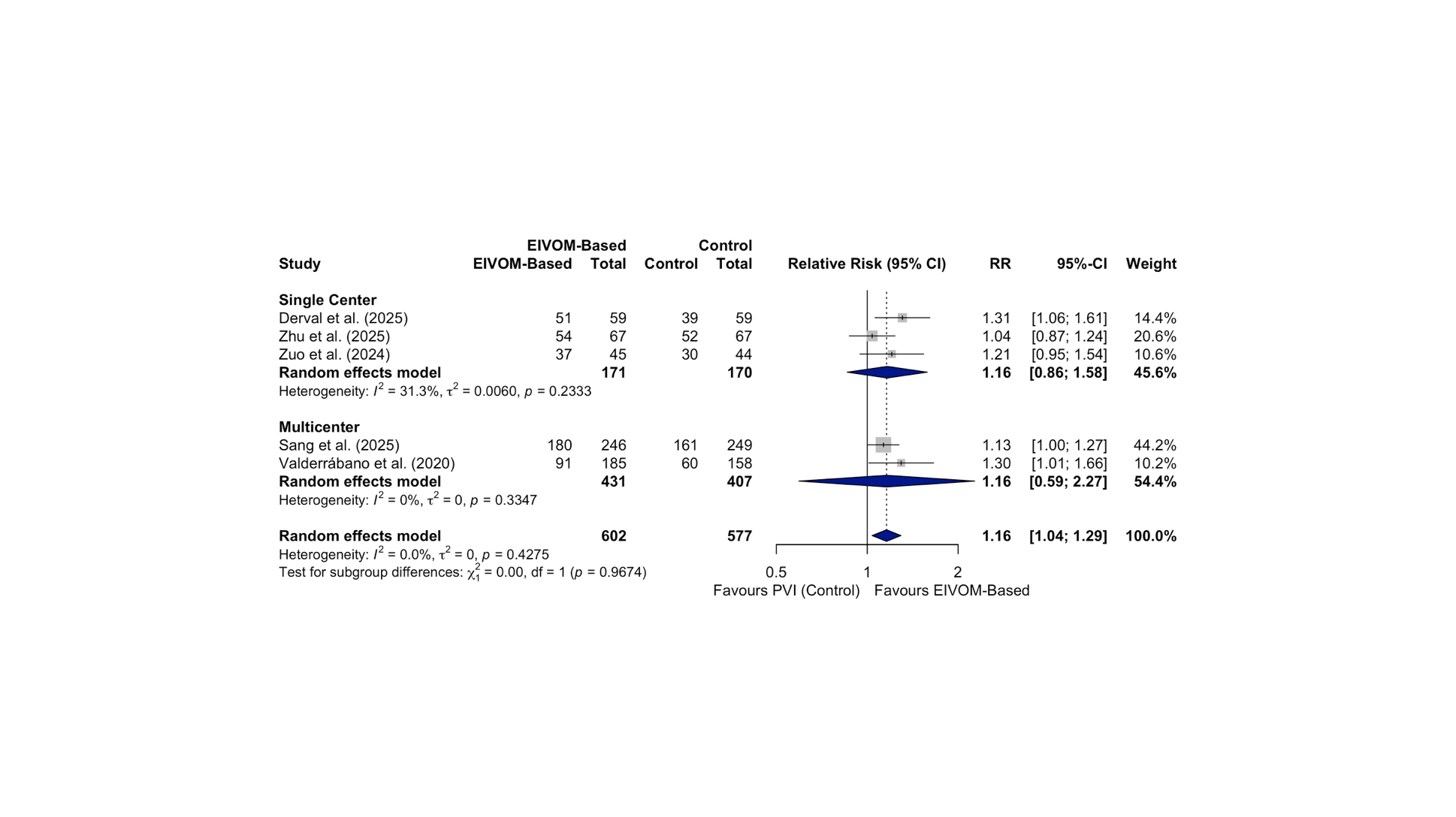
**Supplementary Figure S3:** Subgroup analysis of freedom from any arrhythmia comparing single-center and multicenter RCTs.

**
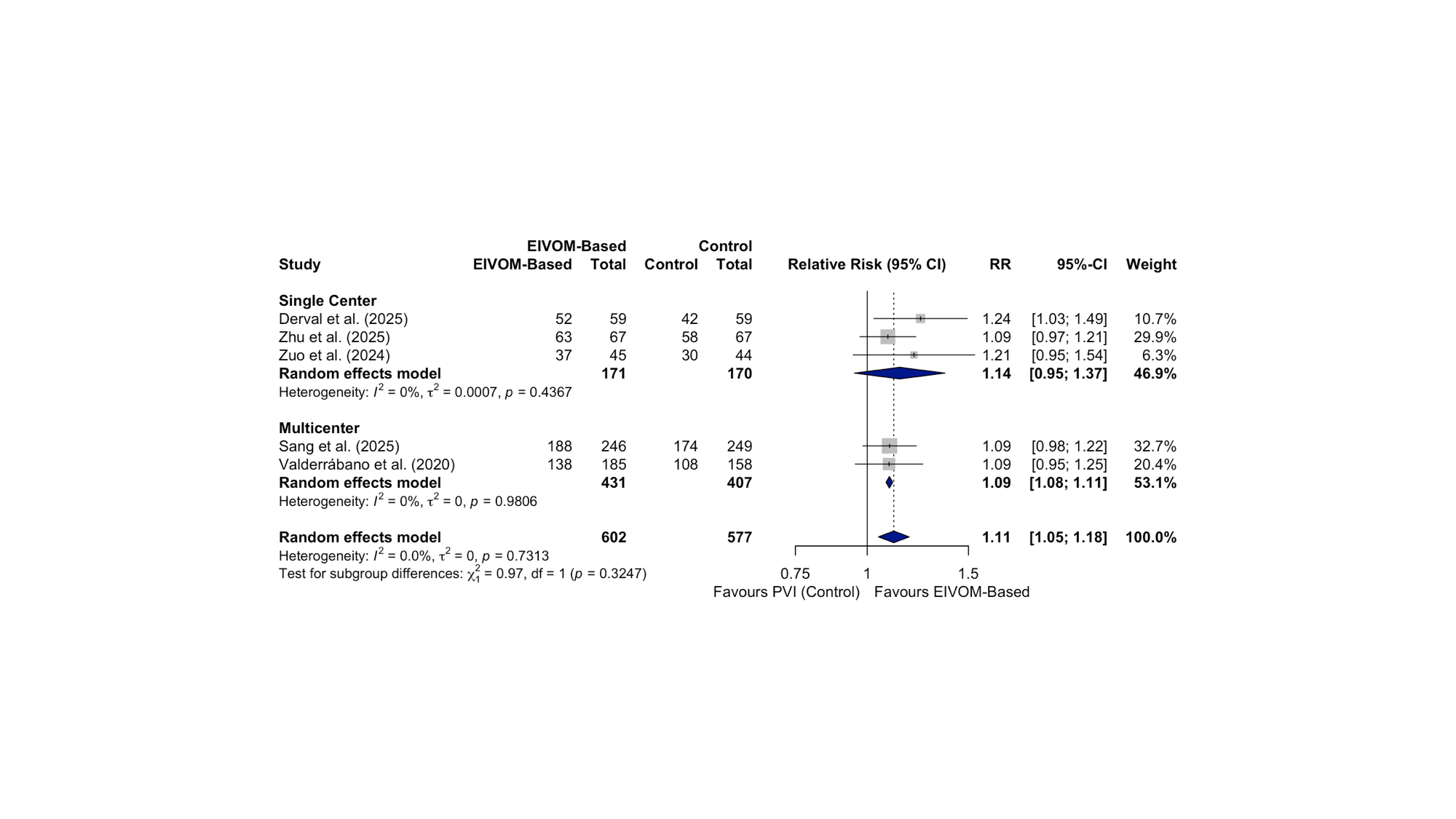
**
**Supplementary Figure S4:** Subgroup analysis of freedom from AF comparing single-center and multicenter RCTs.


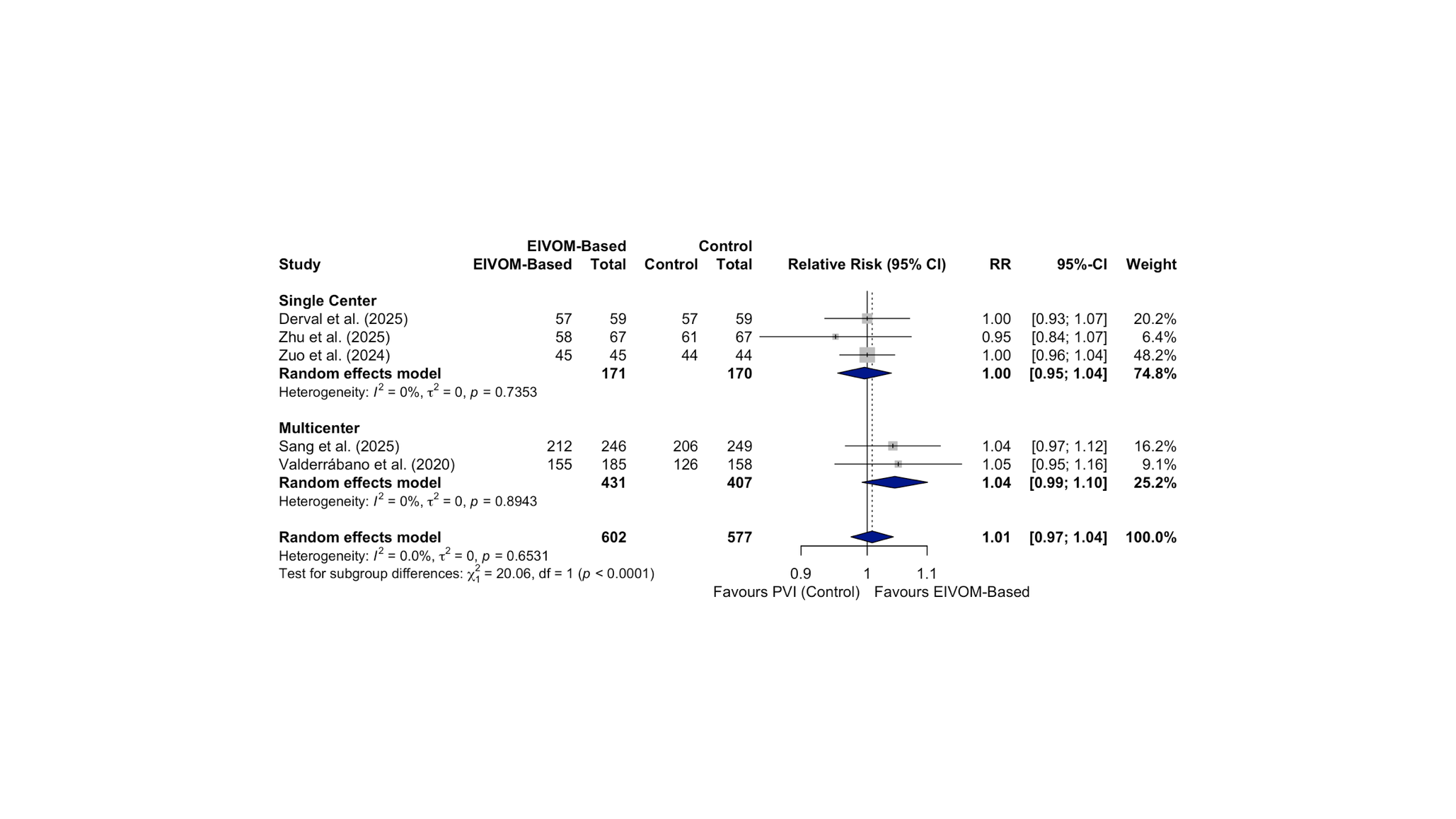
**Supplementary Figure S5:** Subgroup analysis of freedom from AT/AFL comparing single-center and multicenter RCTs.

**Geographic Subgroup Analysis**

To evaluate the generalizability of the intervention and assess for potential regional variance, a prespecified geographic subgroup analysis was conducted, stratifying the included trials into China (n=3) and non-China (n=2) cohorts. For the composite endpoint of freedom from any atrial tachyarrhythmia, formal testing revealed a highly significant subgroup interaction (χ² = 19.93; *P*<0.0001) (**Supplementary figure S6**). The EIVOM-based strategy conferred a pronounced and statistically significant benefit in the non-China cohort (RR 1.30; 95% CI 1.23–1.38; I² = 0.0%). In contrast, the treatment effect was attenuated and did not reach statistical significance within the China cohort (RR 1.12; 95% CI 0.96–1.29; I² = 0.0%). However, for the endpoint of freedom from AF at 12 months, the treatment effect was consistent between the China (RR 1.10; 95% CI 1.01–1.20; I² = 0.0%) and Non-China (RR 1.14; 95% CI 0.53–2.47; I² = 12.7%) cohorts, with no significant subgroup interaction (χ² = 0.34; *P*=0.56) (**Supplementary figure S7**). Similarly, freedom from AT/AFl demonstrated uniform treatment effects across regions without significant subgroup heterogeneity (χ² = 0.14; *P*= 0.71) (**Supplementary figure S8**). These data indicate that while the EIVOM-based strategy demonstrates consistent efficacy regarding primary AF suppression, the absolute magnitude of protection against total atrial arrhythmias is significantly influenced by regional cohort characteristics, patient selection, or adjunctive procedural workflows.


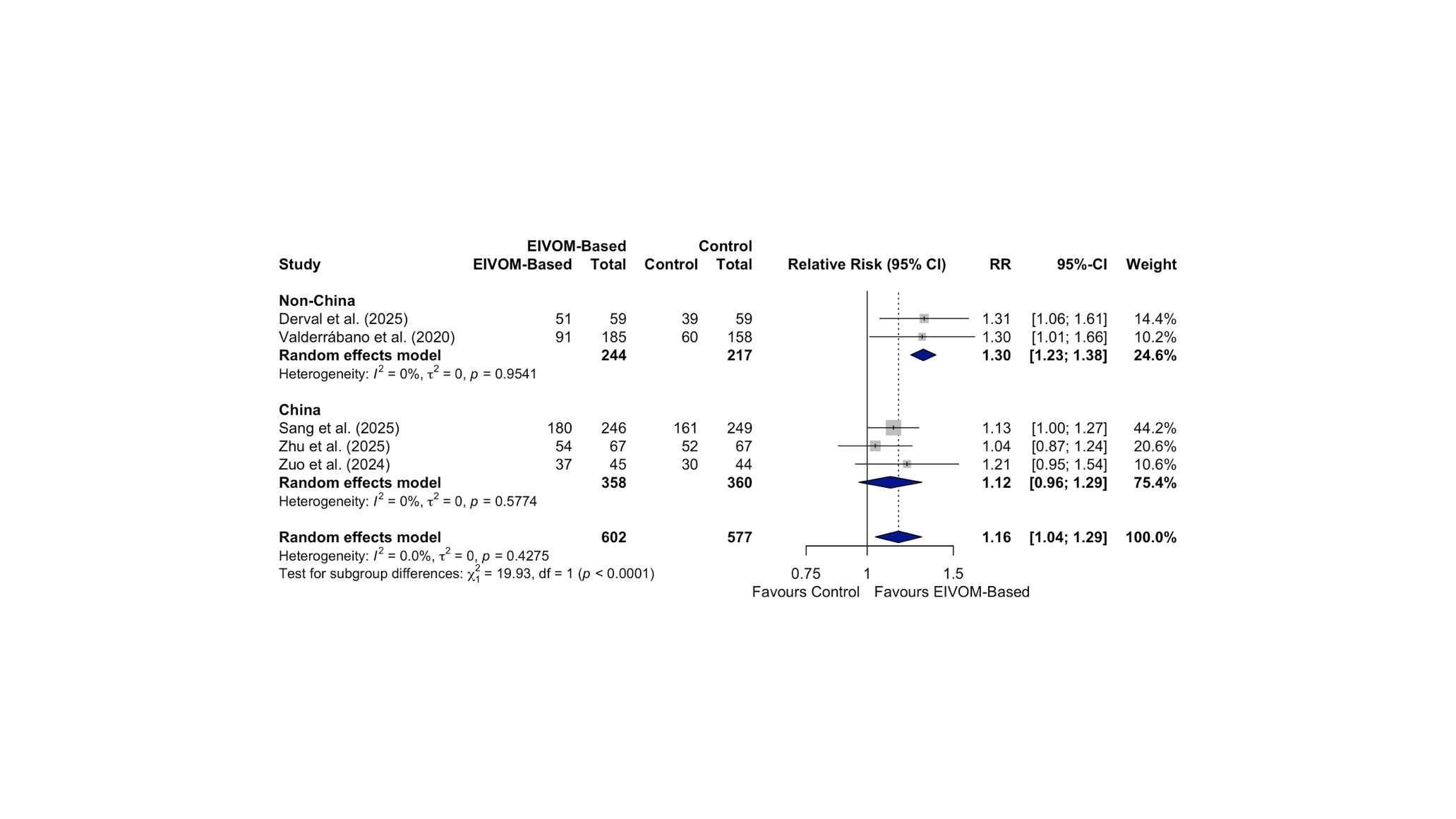
**Supplementary Figure S6**: Geographic subgroup analysis of freedom from any atrial arrhythmia at 12 months.


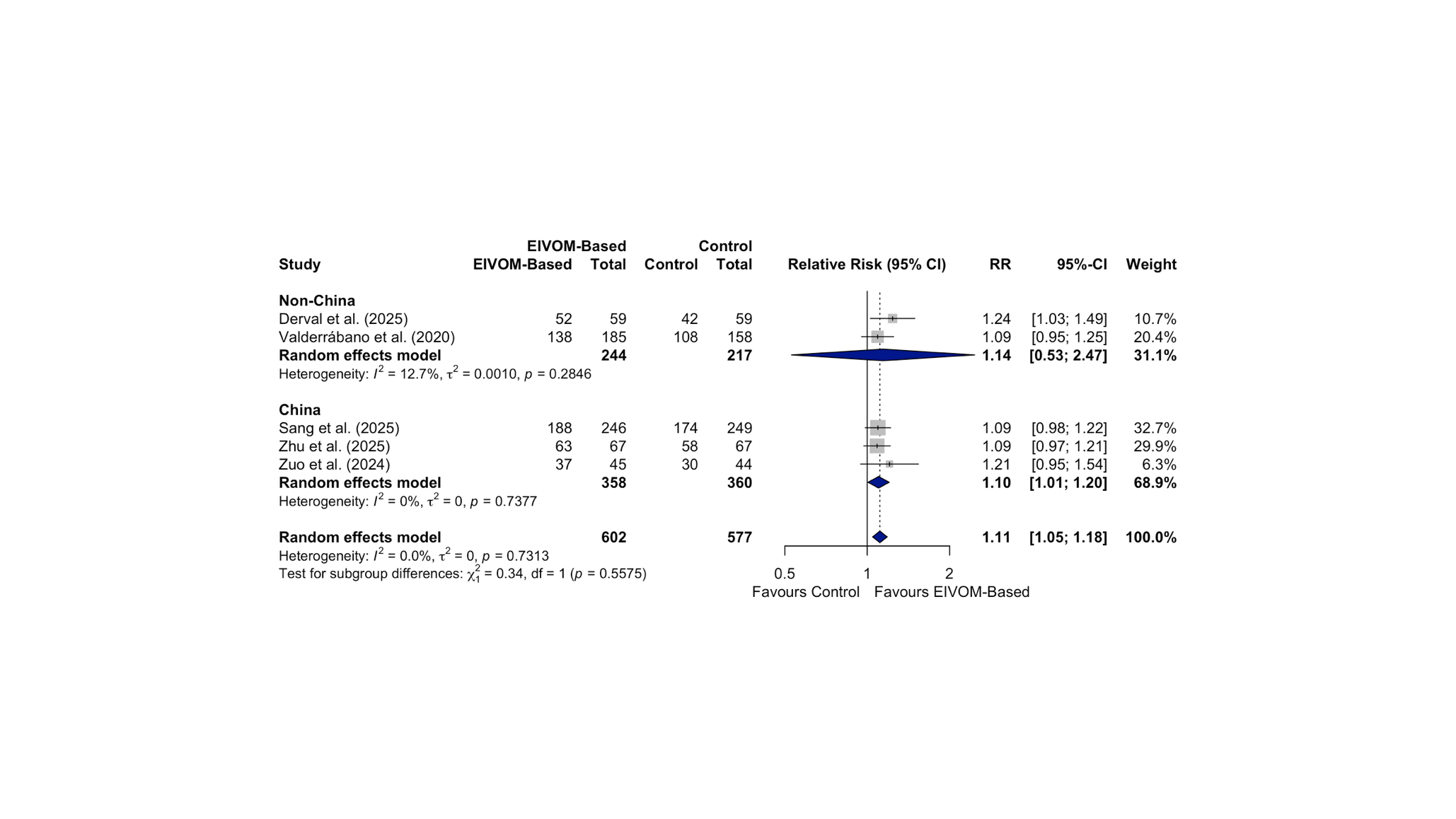
**Supplementary Figure S7**: Geographic subgroup analysis of freedom from atrial fibrillation at 12 months.

**Supplementary Figure S8**: Geographic subgroup analysis of freedom from atrial tachycardia or atrial flutter at 12 months.


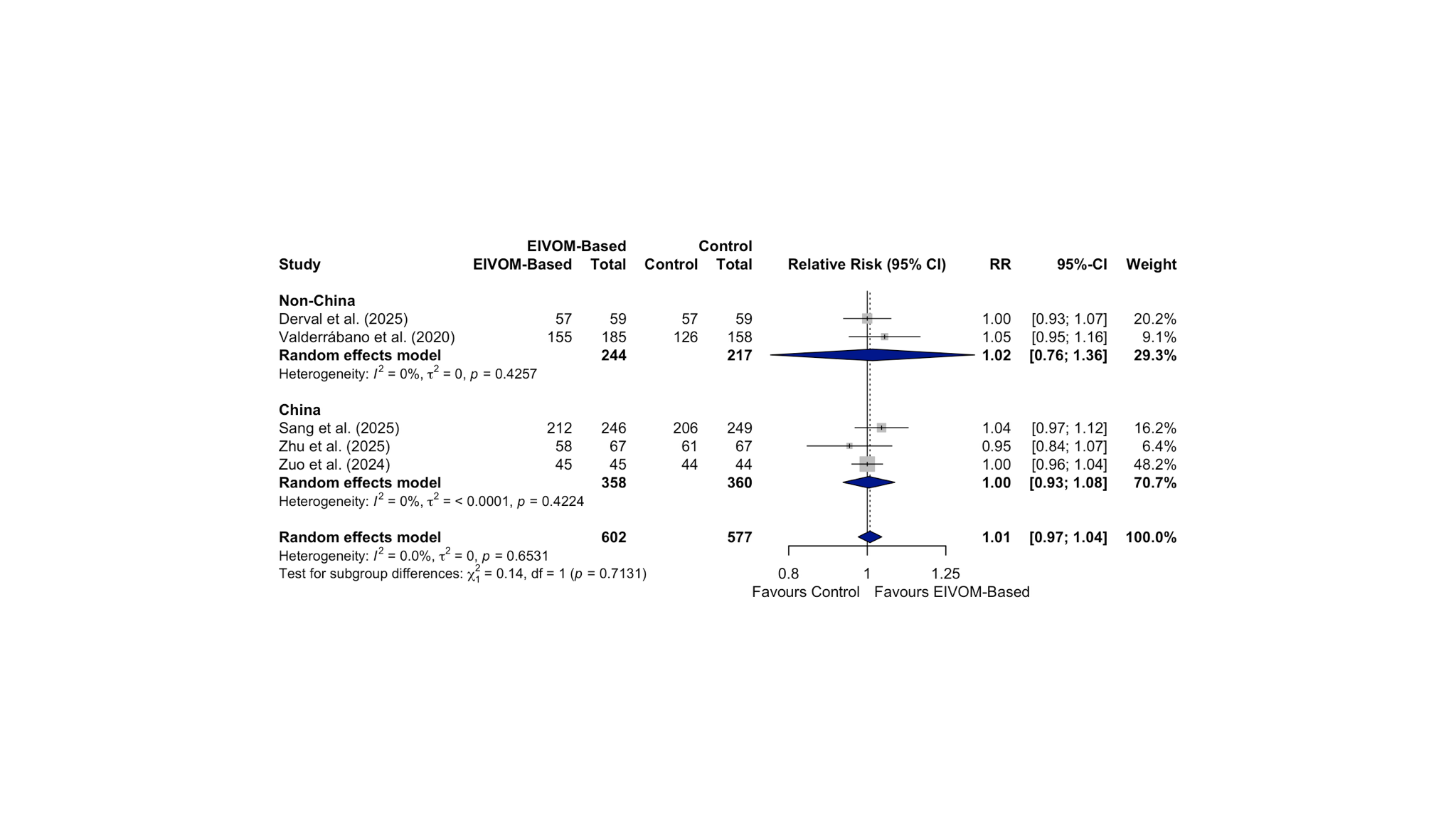


**
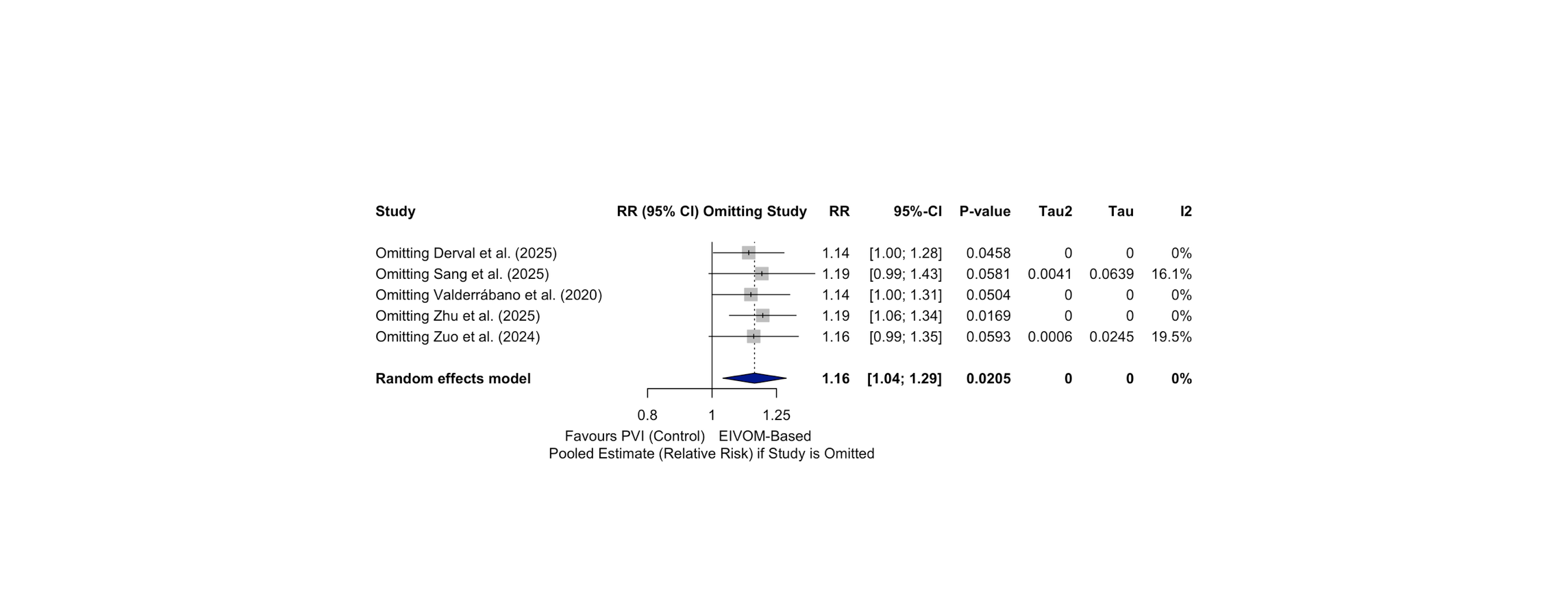
Supplementary Figure S9**: Leave-one-out sensitivity analysis for freedom from any arrhythmia.


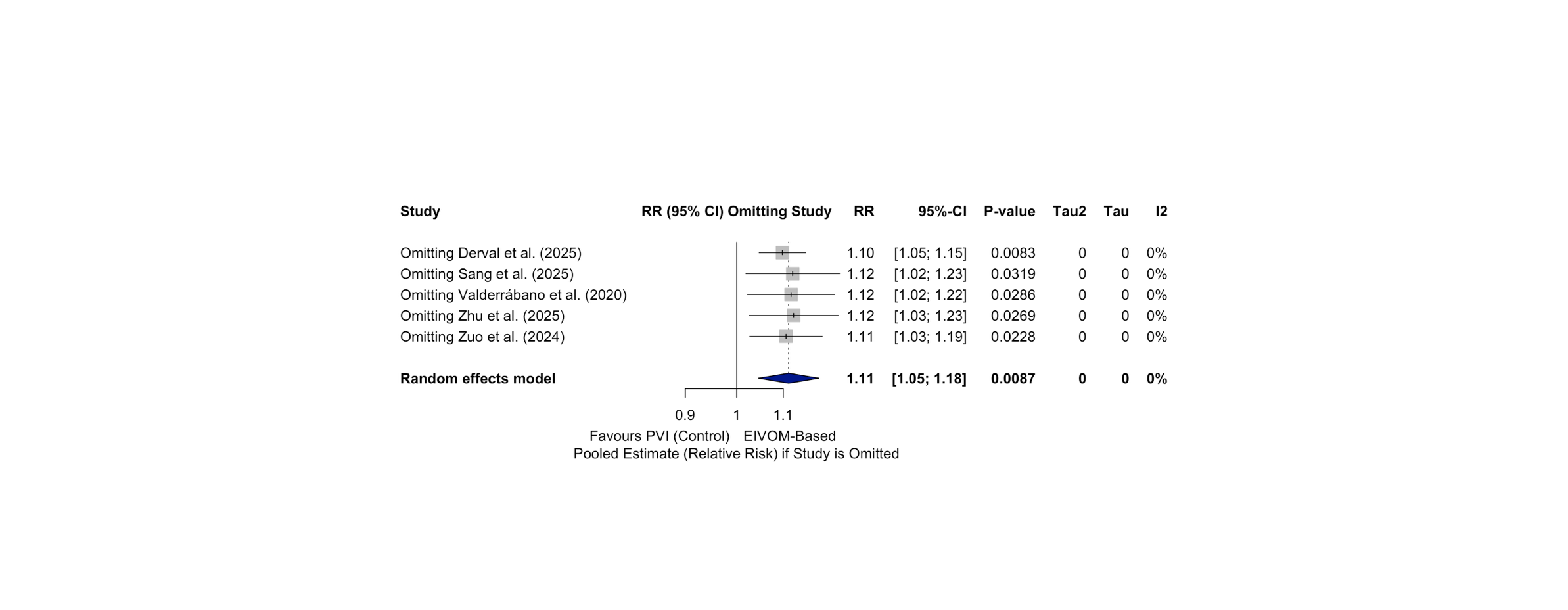
**Supplementary Figure S10**: Leave-one-out sensitivity analysis for freedom from AF.

**Supplementary Figure S11**: Leave-one-out sensitivity analysis for freedom from AT/AFL.


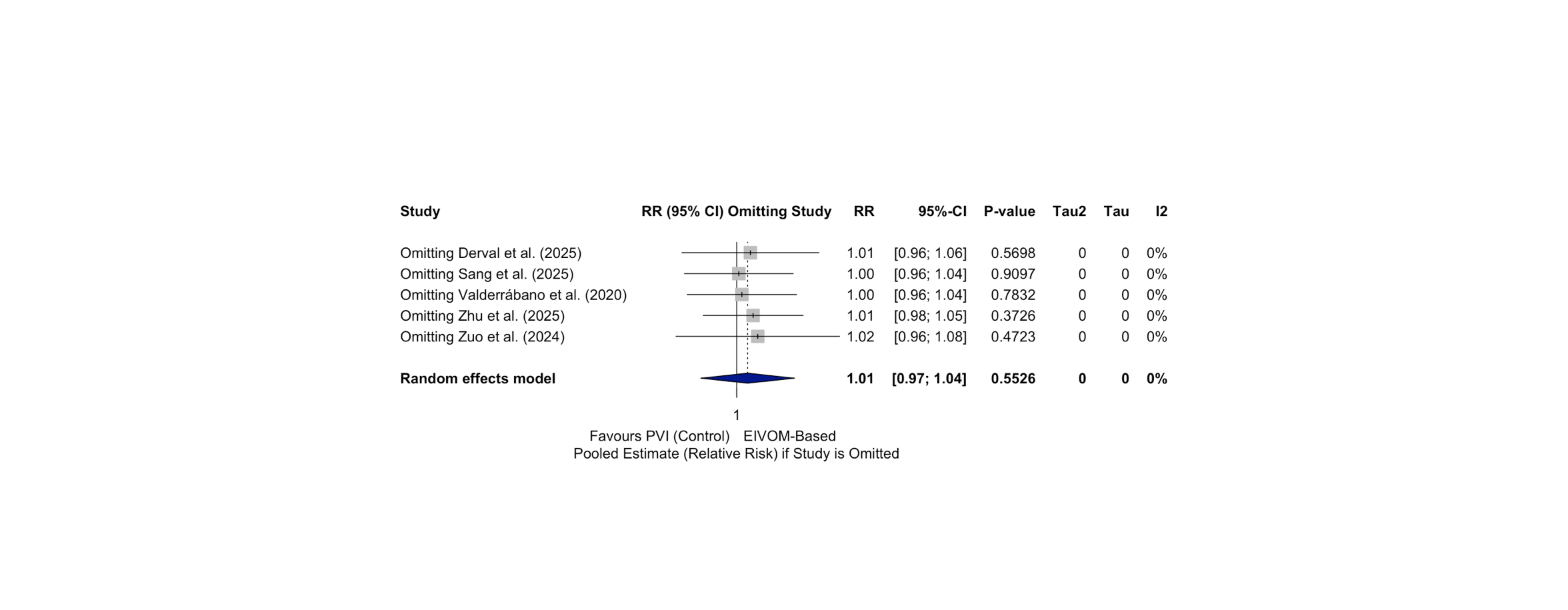


**Supplementary Figure S12**: Leave-one-out sensitivity analysis for procedural time.

**
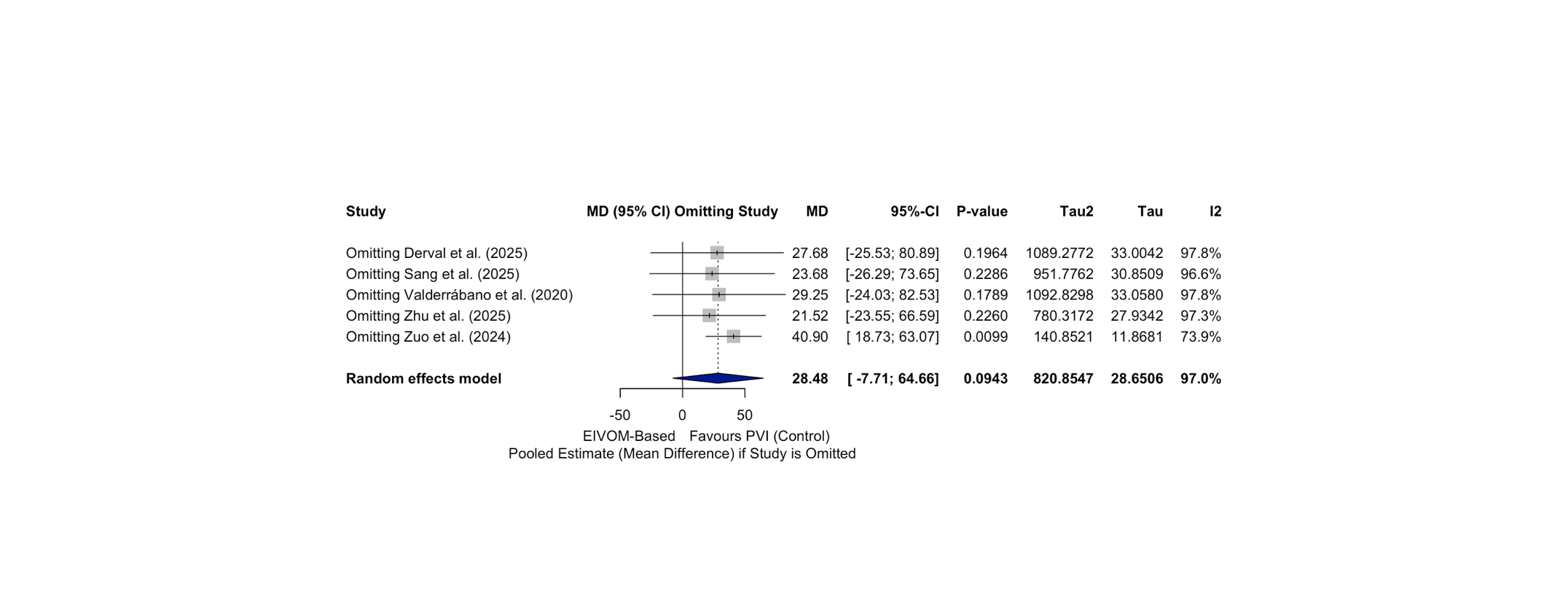
**

**Supplementary Figure S13**: Leave-one-out sensitivity analysis for fluoroscopy time.


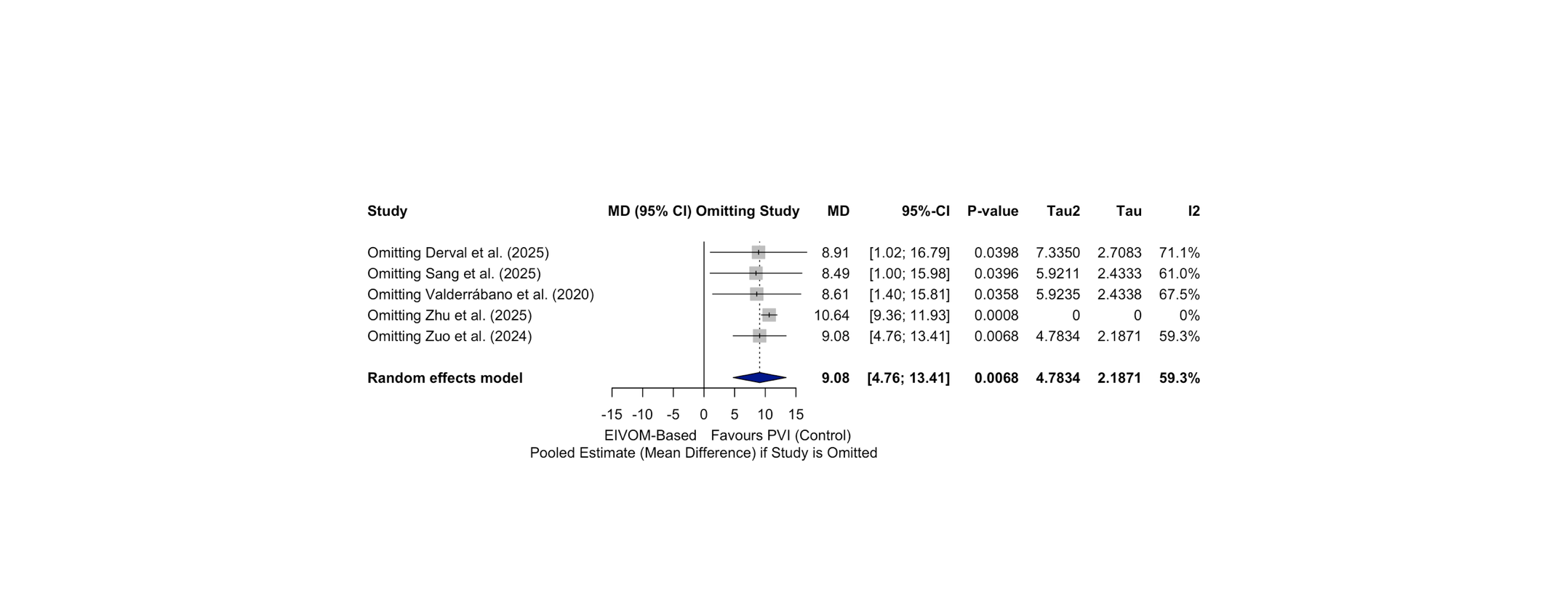


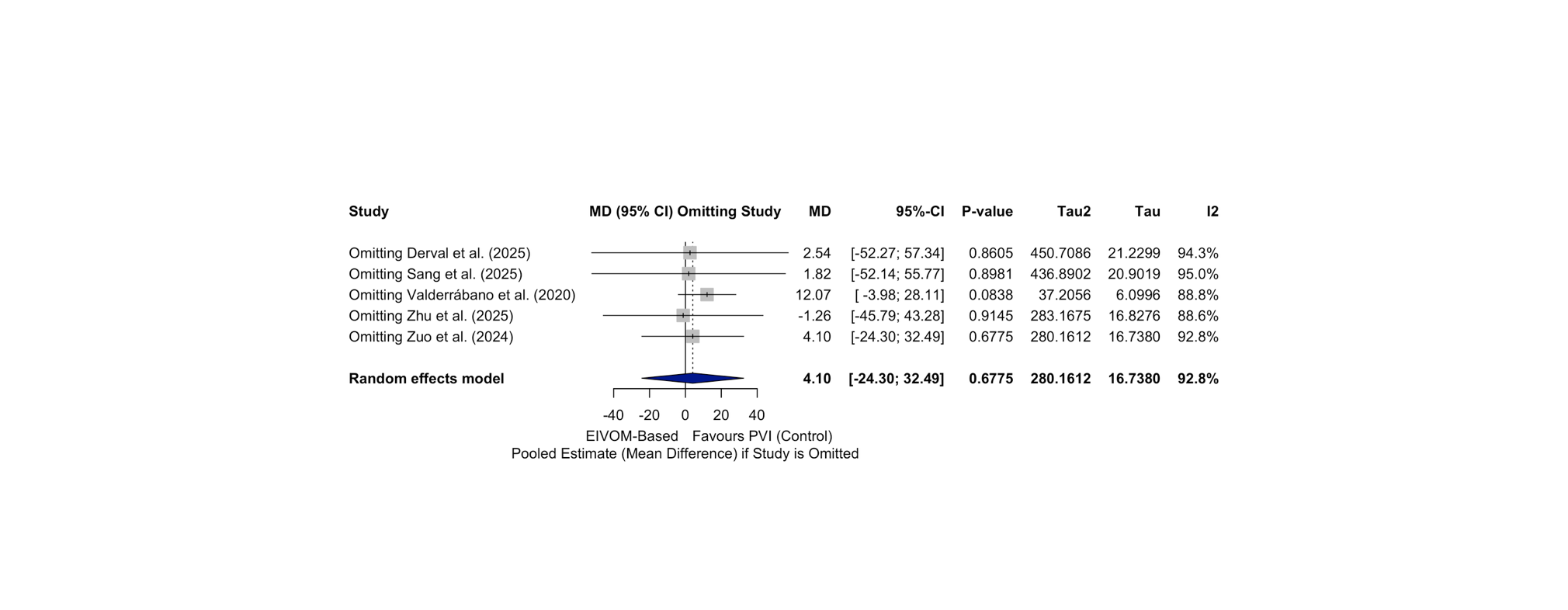
**Supplementary Figure S14**: Leave-one-out sensitivity analysis for ablation time.


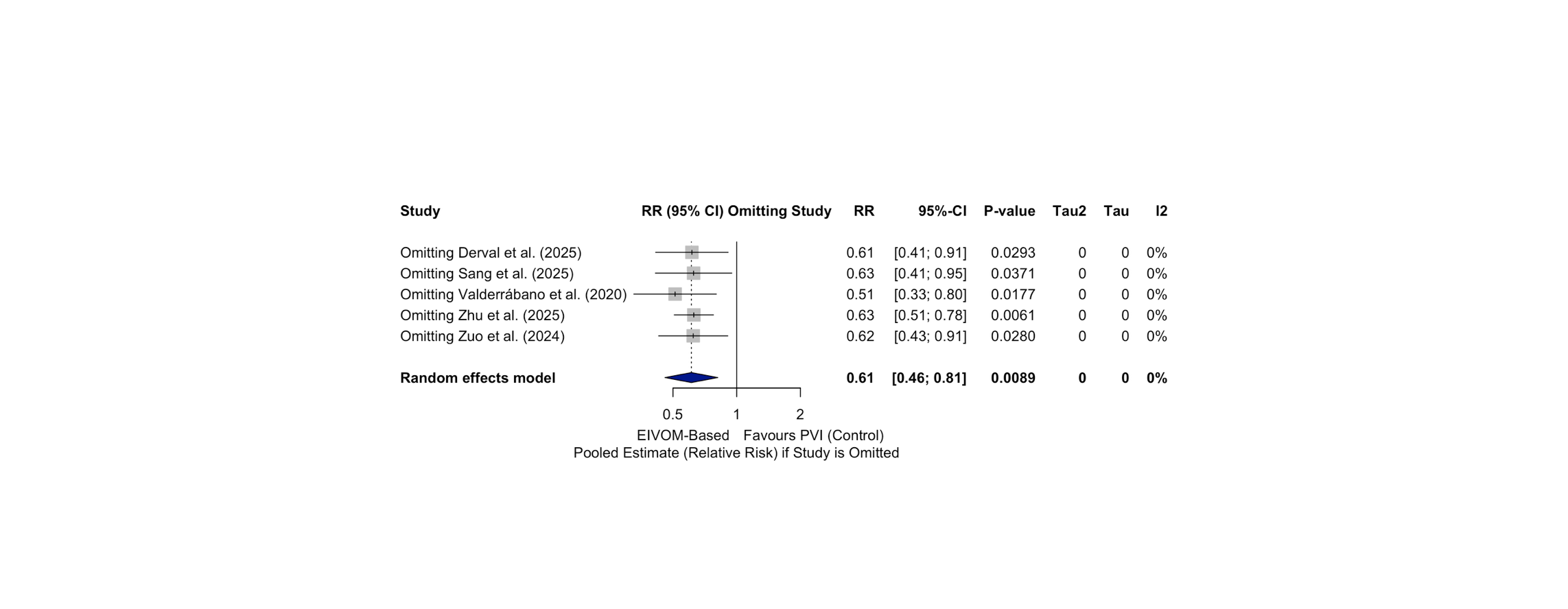
**Supplementary Figure S15**: Leave-one-out sensitivity analysis for repeat ablation.

**
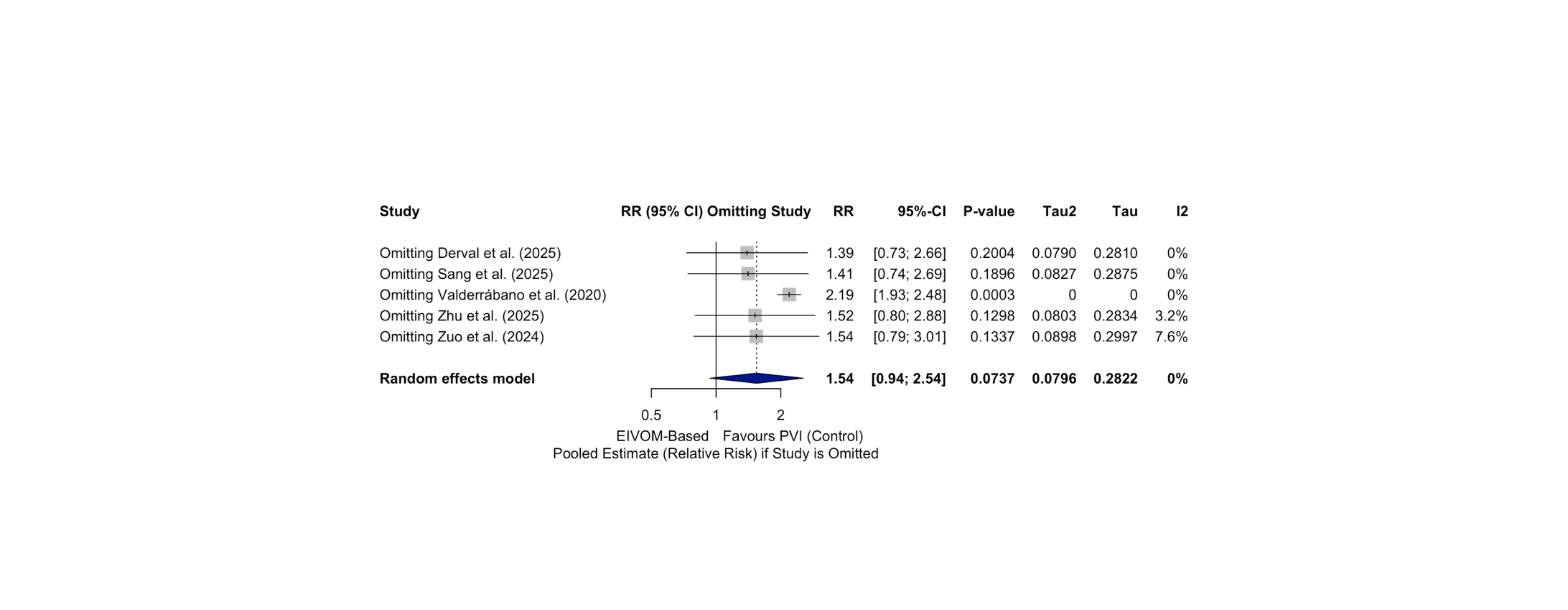
Supplementary Figure S16**: Leave-one-out sensitivity analysis for any complications.


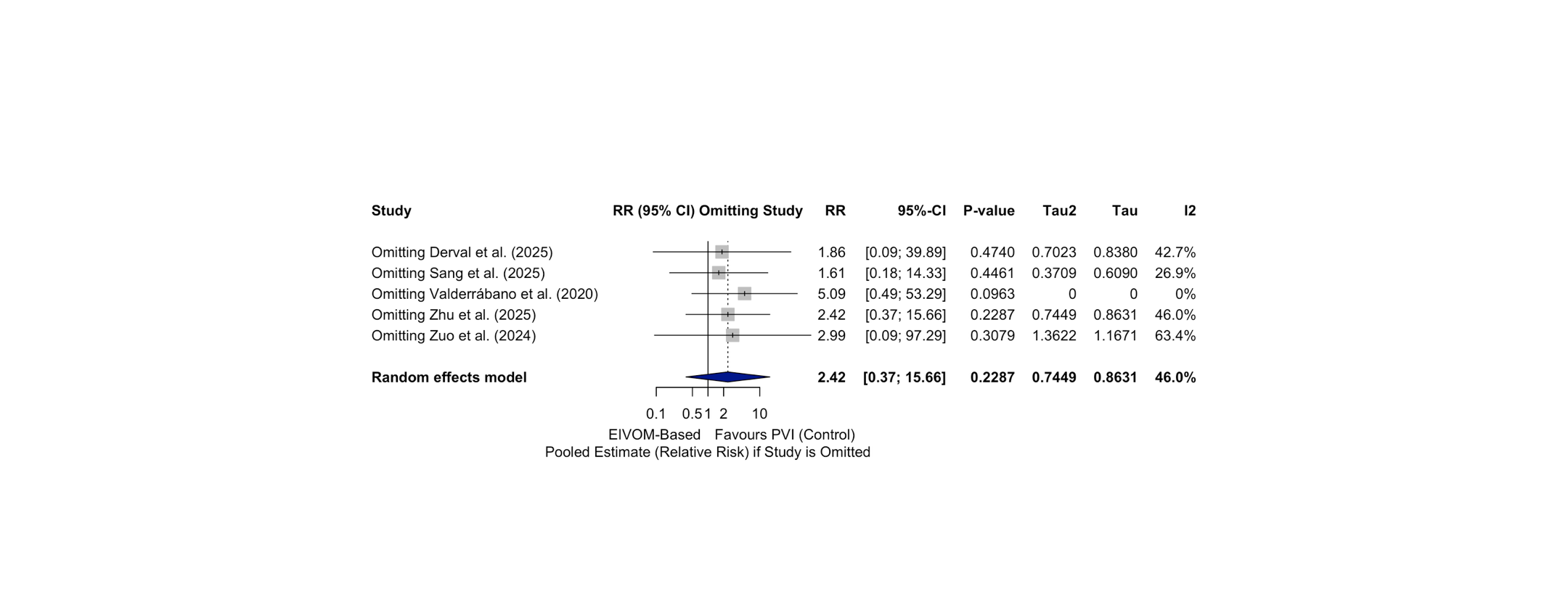
**Supplementary Figure S17**: Leave-one-out sensitivity analysis for pericardial effusion/pericarditis.


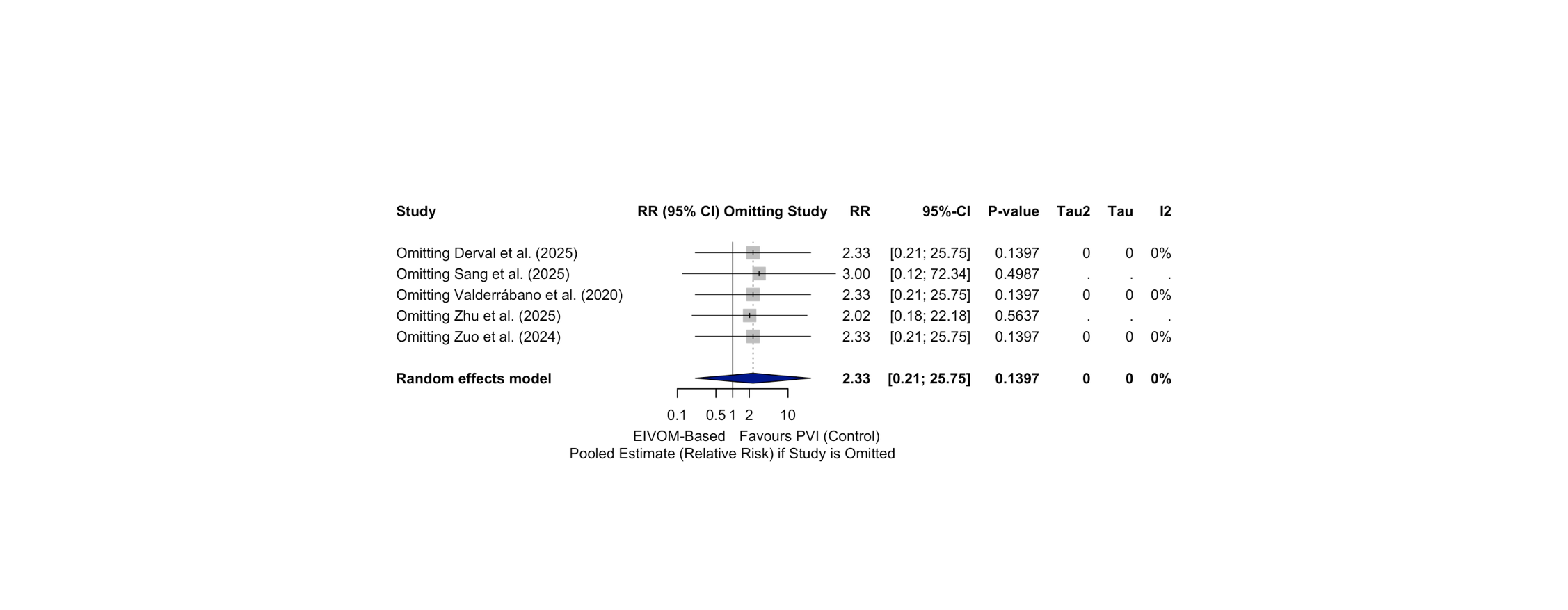

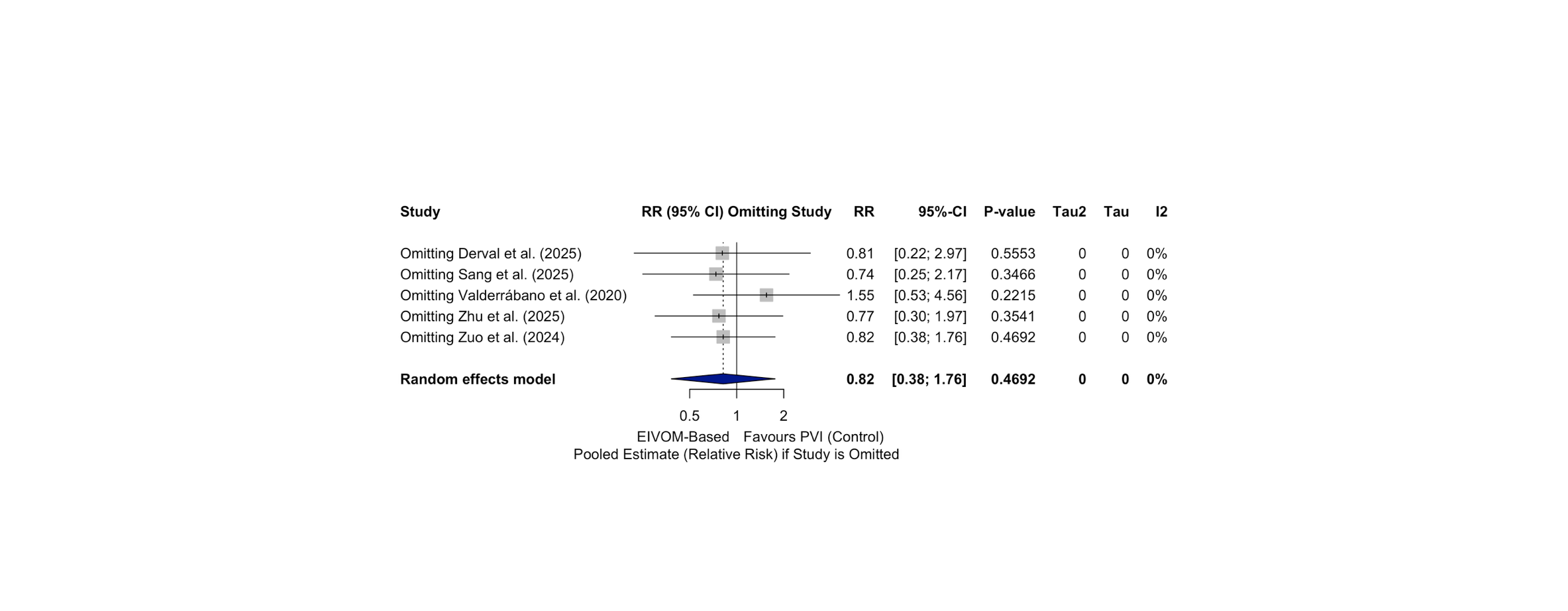
**Supplementary Figure S18**: Leave-one-out sensitivity analysis for major complications.

**Supplementary Figure S19**: Leave-one-out sensitivity analysis for cardiac tamponade.


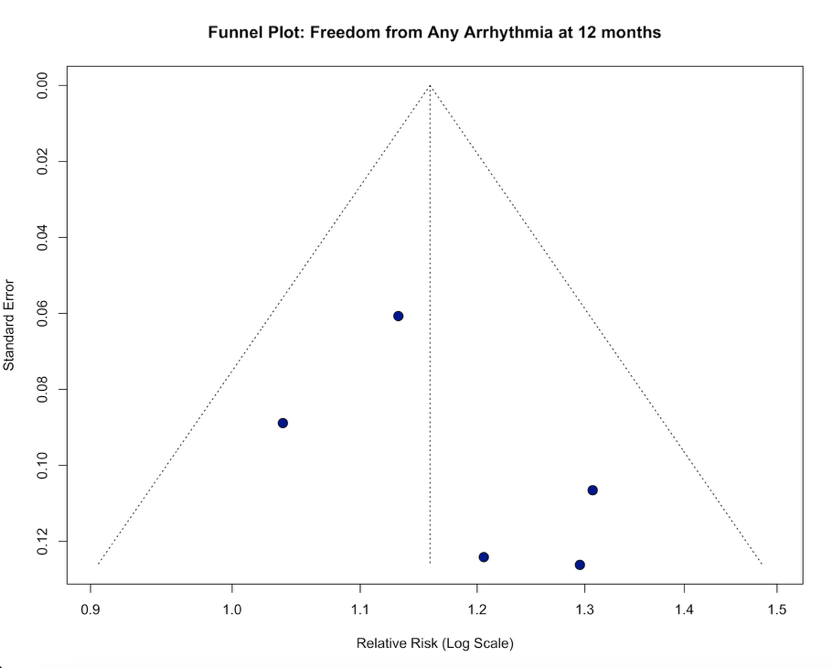
**Supplementary Figure S20**: Funnel plot for freedom from any arrhythmia at 12 months.

**Supplementary Figure S21**: Funnel plot for freedom from atrial fibrillation at 12 months.


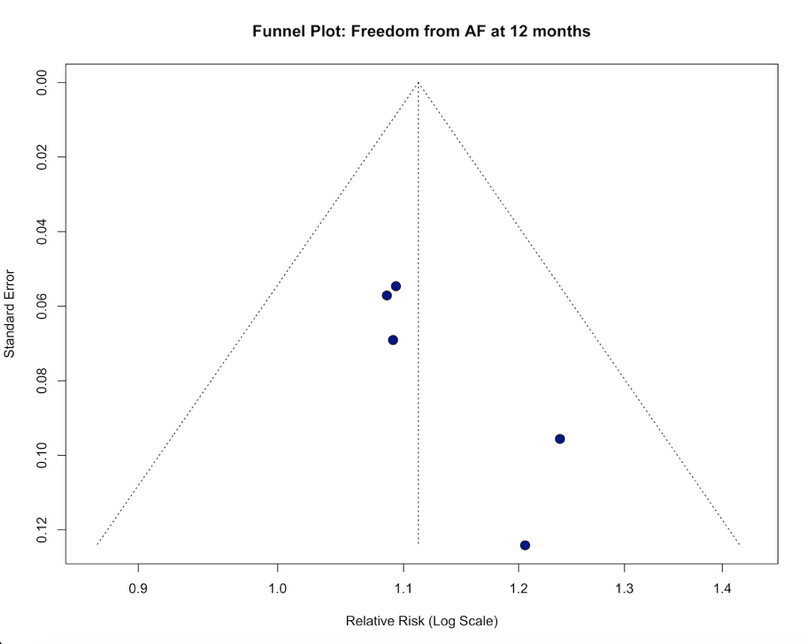


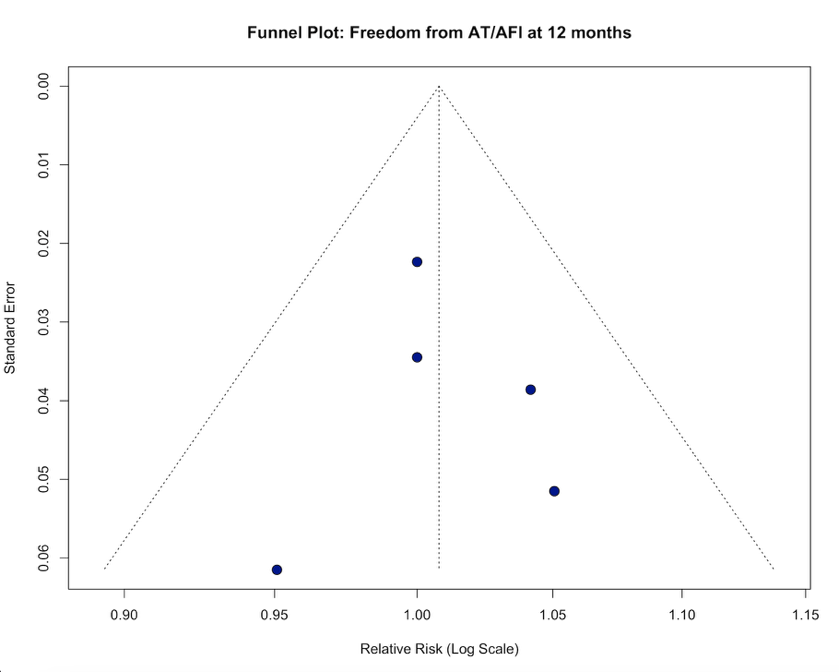
**Supplementary Figure S22**: Funnel plot for freedom from AT/AFL.

**Supplementary Table S3:** Simple co-variate meta-regression.

| Outcome | Covariate | Coefficient | SE | P-Value | Significance |
| --- | --- | --- | --- | --- | --- |
| Freedom from AF | Mean Age | 0.007 | 0.013 | 0.579 | ns |
|  | Female Sex (%) | 0.001 | 0.006 | 0.908 | ns |
|  | LA Diameter (mm) | -0.007 | 0.021 | 0.753 | ns |
|  | AF Duration (mo) | -0.001 | 0.007 | 0.888 | ns |
|  | LVEF (%) | -0.005 | 0.009 | 0.549 | ns |
|  | CHA2DS2-VASc | 0.013 | 0.049 | 0.784 | ns |
| Any Arrhythmia | Mean Age | 0.035 | 0.019 | 0.063 | ns |
|  | Female Sex (%) | -0.001 | 0.006 | 0.925 | ns |
|  | LA Diameter (mm) | -0.010 | 0.029 | 0.729 | ns |
|  | AF Duration (mo) | -0.004 | 0.008 | 0.627 | ns |
|  | LVEF (%) | -0.020 | 0.012 | 0.096 | ns |
|  | CHA2DS2-VASc | 0.097 | 0.072 | 0.178 | ns |
| Repeat Ablation | Mean Age | 0.064 | 0.072 | 0.380 | ns |
|  | Female Sex (%) | -0.011 | 0.030 | 0.715 | ns |
|  | LA Diameter (mm) | 0.061 | 0.123 | 0.622 | ns |
|  | AF Duration (mo) | -0.012 | 0.038 | 0.755 | ns |
|  | LVEF (%) | -0.036 | 0.047 | 0.443 | ns |
|  | CHA2DS2-VASc | 0.174 | 0.231 | 0.453 | ns |
| Any Complications | Mean Age | -0.137 | 0.095 | 0.151 | ns |
|  | Female Sex (%) | -0.002 | 0.052 | 0.969 | ns |
|  | LA Diameter (mm) | -0.221 | 0.168 | 0.189 | ns |
|  | AF Duration (mo) | -0.001 | 0.063 | 0.985 | ns |
|  | LVEF (%) | 0.074 | 0.068 | 0.277 | ns |
|  | CHA2DS2-VASc | -0.479 | 0.292 | 0.101 | ns |

**Trial Sequential Analysis**

Trial sequential analysis (TSA) was performed for the primary efficacy endpoints to assess conclusiveness and risk of random error. TSA was exploratory for safety endpoints. Due to the low incidence of adverse events (e.g., major complication rate ~2.8%), the required information size (RIS) to detect a 25% relative risk increase (RRI) was excessively large (N > 7,000 for any complications; N >19,000 for major complications). Consequently, TSA figures for safety outcomes were deemed uninformative for the main manuscript and are omitted (**Supplementary Table S4**). Given the low event rates for safety endpoints, heterogeneity estimates are unstable. We utilized a diversity adjustment (D^2^) of 0% to calculate the absolute minimum RIS. Even under this conservative assumption of homogeneity, the required sample size remains unattainable with current data. TSA was not performed for continuous procedural outcomes, including total procedural time, fluoroscopy time, and ablation time due to substantial statistical heterogeneity. This high variance renders the diversity-adjusted RIS statistically unstable. Furthermore, unlike clinical adverse events, there are no established safety or efficacy monitoring boundaries that would necessitate premature trial termination based exclusively on procedural duration.

**Supplementary Table S4**: Trial sequential analysis of safety endpoints.

| **Parameter** | **Alpha, Power (%)** | **Control Rate (%)** | **Relative Risk Increase (%)** | **D^2^ (%)** | **Required Information Size (RIS)** |
| --- | --- | --- | --- | --- | --- |
| **Any Complications** | $\alpha$ = 5%, power = 80% | 6.9 | 25 | 0 | 7,517 |
| **Major Complications** | $\alpha$ = 5%, power = 80% | 2.8 | 25 | 0 | 19,744 |
| **Pericardial Effusion/Pericarditis** | $\alpha$ = 5%, power = 80% | 2.6 | 25 | 0 | 21,103 |
| **Cardiac Tamponade** | $\alpha$ = 5%, power = 80% | 0.17 | 25 | 0 | 325,439 |

**
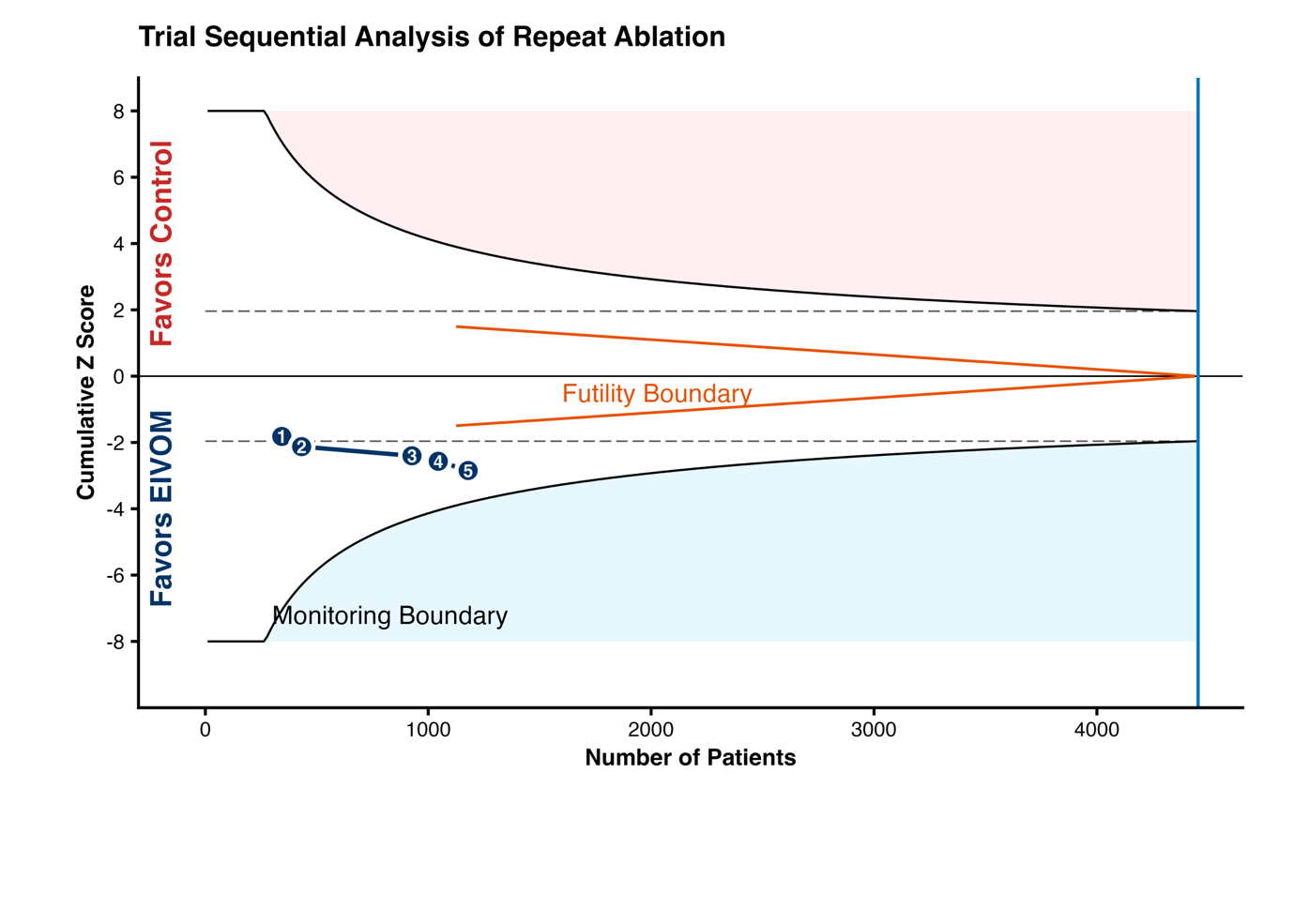
Supplementary Figure S23**: Trial Sequential Analysis for Repeat Ablation.

**Repeat Ablation**: ($\alpha$=5%, Power=80%, Control Rate=13.9%, RRR=20%, D^2^=0% (RIS=4,454))

For repeat ablation, conventional meta-analysis indicated a statistically significant benefit favoring EIVOM (Z > 1.96). However, the TSA remained inconclusive; the cumulative Z-curve did not cross the trial sequential monitoring boundary, and the accrued information size (N = 1,179) was well below the required information size (N=4,454). This discrepancy is attributable to the low incidence of repeat ablation in the control group (13.9%), which necessitates a larger sample size to confirm a 20% relative risk reduction definitively.
